# Identification, Characterization and Expression Profiling of Stress-Related Genes in Easter Lily (*Lilium formolongi*)

**DOI:** 10.3390/genes8070172

**Published:** 2017-06-27

**Authors:** Jewel Howlader, Jong-In Park, Arif Hasan Khan Robin, Kanij Rukshana Sumi, Ill-Sup Nou

**Affiliations:** 1 Department of Horticulture, Sunchon National University, 255, Jungang-ro, Suncheon, Jeonnam 57922, Korea; jewel.howlader81@gmail.com (J.H.); jipark@sunchon.ac.kr (J.-I.P.); gpb21bau@gmail.com (A.H.K.R.); 2 Department of Fisheries Science, Chonnam National University, 50, Daehak-ro, Yeosu, Jeonnam 59626, Korea; krsumi@pstu.ac.bd

**Keywords:** abiotic stress, *Botrytis* inoculation, stress-related genes, RACE-PCR, *Lilium formolongi*

## Abstract

Biotic and abiotic stresses are the major causes of crop loss in lily worldwide. In this study, we retrieved 12 defense-related expressed sequence tags (ESTs) from the NCBI database and cloned, characterized, and established seven of these genes as stress-induced genes in *Lilium formolongi*. Using rapid amplification of cDNA ends PCR (RACE-PCR), we successfully cloned seven full-length mRNA sequences from *L. formolongi* line Sinnapal lily. Based on the presence of highly conserved characteristic domains and phylogenetic analysis using reference protein sequences, we provided new nomenclature for the seven nucleotide and protein sequences and submitted them to GenBank. The real-time quantitative PCR (qPCR) relative expression analysis of these seven genes, including *LfHsp70-1*, *LfHsp70-2*, *LfHsp70-3*, *LfHsp90*, *LfUb*, *LfCyt-b5*, and *LfRab*, demonstrated that they were differentially expressed in all organs examined, possibly indicating functional redundancy. We also investigated the qPCR relative expression levels under two biotic and four abiotic stress conditions. All seven genes were induced by *Botrytis cinerea* treatment, and all genes except *LfHsp70-3* and *LfHsp90* were induced by *Botrytis elliptica* treatment; these genes might be associated with disease tolerance mechanisms in *L. formolongi*. In addition, *LfHsp70-1*, *LfHsp70-2*, *LfHsp70-3*, *LfHsp90*, *LfUb*, and *LfCyt-b5* were induced by heat treatment, *LfHsp70-1*, *LfHsp70-2*, *LfHsp70-3*, *LfHsp90*, and *LfCyt-b5* were induced by cold treatment, and *LfHsp70-1*, *LfHsp70-2*, *LfHsp70-3*, *LfHsp90*, *LfCy-b5*, and *LfRab* were induced by drought and salt stress, indicating their likely association with tolerance to these stress conditions. The stress-induced candidate genes identified in this study provide a basis for further functional analysis and the development of stress-resistant *L. formolongi* cultivars.

## 1. Introduction

Lily (*Lilium* L., 2*n* = 2*x* = 24), comprising members of the Liliaceae family, is one of the most popular groups of ornamental bulbous monocot outcrossing perennial herbs worldwide due to their incomparable beauty and commercial importance [1]. The *Lilium* genus contains nearly 110 to 115 species, which are primarily distributed in the cold and temperate regions of the Northern Hemisphere (10° N to 60° N), particularly Asia, North America, and Europe [2,3]. Among Asian countries, China, Nepal, Korea, and Japan are renowned centers of diversity of this genus worldwide [4]. Lily is one of the most important cut flowers in Korea. In 2011, the total cultivation area of Korean lily was 215 ha in the major growing regions of Gangwon, Jeju, and Chungcheongnam provinces with an annual production value of US$34 million according to the Ministry of Agriculture, Food, and Rural Affairs of the Republic of Korea [5]. The main export market of Korean cut lilies is Japan, totaling more than US$3 million in 2012 [6], but these flowers are also exported to the United States, China, and The Netherlands.

Like many other crops, lily faces a wide range of abiotic and biotic stresses. For example, substantial economic losses in lily production are attributed to leaf blight caused by *Botrytis* infection [7,8,9]. In addition, abiotic stresses, such as high and low temperature, drought, and salinity cause considerable degradation of lily flower quality and severely affect plant growth and development, resulting in reduced total production worldwide [10,11,12]. Furthermore, the ecological distribution of naturally grown Korean *Lilium spp.* has changed due to the adverse effects of climate change, resulting in a gradual decline in *Lilium* germplasm resources [13]. Therefore, it is important to develop lily cultivars with biotic and abiotic stress resistance to protect and conserve lily resources worldwide.

Efforts to explore potential resistance genes have helped speed the development of plant varieties with resistance to *Botrytis spp.*, as well as to various abiotic stresses. Expressed sequence tag (EST) analysis provides a basis for selecting resistance-related genes [14]. Previously, published functional role categories of defense-related ESTs from *L*. *longiflorum* generative cells are available for further analysis [15]. ESTs are incomplete, unedited, randomly selected single-pass sequences derived from complementary DNA (cDNA) libraries and are highly valuable for further molecular analysis [16]. Rapid amplification of cDNA ends-PCR (RACE-PCR) can be used to obtain the full-length cDNA (mRNA) and protein sequences of stress-induced candidate EST genes [14].

Plants contain several types of stress-induced proteins, including HSP70 proteins, HSP90 proteins, ubiquitin proteins, cytochrome-b5 heme/steroid binding proteins, and RAB domain containing proteins. HSPs play an important role in maintaining cellular homeostasis under normal and various stress conditions in both prokaryotic and eukaryotic cells [17]. Ubiquitin proteins function through the ubiquitination of other proteins, especially HSPs. Under stress conditions, plants increase their ubiquitination capacity, which affects hormone biosynthesis, hormonal signaling cascades, and plant defense mechanisms [18,19]. In addition, cytochrome-*b5* acts as an electron-transfer component in the desaturation reaction [20], which alters membrane fluidity, thereby enhancing adaptation to the environment [21]. Another type of stress-induced protein, the RAB, is conserved from yeast to animals. These proteins are linked to intracellular vesicle trafficking and play a vital role in plant resistance to pathogen and environmental stresses [22,23].

Little is known about biotic and abiotic stress-induced resistance genes in *Lilium formolongi*, whereas various molecular techniques, especially EST analysis, have been widely used to identify genes that function in biotic and abiotic stress responses in other plant species [24,25,26,27]. In the current study, we identified full-length stress-related candidate genes using ESTs from *L. formolongi* under various stress conditions to facilitate the development of stress-resistant *L. formolongi* varieties. We validated the stress-induced candidate genes through real-time quantitative PCR (qPCR) expression analysis of the stress-treated samples. Finally, we analyzed the deduced protein and nucleotide sequences of these genes in silico. This study lays the foundation for future plant breeding studies of stress resistance genes in the Liliaceae family.

## 2. Materials and Methods

### 2.1. Defense-Related EST Sequence Retrieval

The sequences of 12 defense-related EST genes identified from EST sequence analysis from *L. longiflorum* generative cells [15] were obtained from the National Center for Biotechnology Information (NCBI) database [28] and subjected to nucleotide BLAST searches using the Basic Local Alignment Search Tool (BLAST) from NCBI [29] to detect similarity with defense-related genes from other crop species.

### 2.2. Plant Materials

Seeds of *L. formolongi* line Sinnapal lily, which is susceptible to *Botrytis spp.*, were germinated in plastic pots filled with disinfected soil mixture. The seedlings were grown in growth chambers at 22 °C under a 16 h light/8 h dark photoperiod with a photon flux density of 140 μmol m^−2^ s^−1^ at the Department of Horticulture, Sunchon National University, South Korea for both biotic and abiotic stress treatments. The relative humidity was maintained between 65% and 75%. Fresh roots, stems, leaves at the 5–6 leaf stage (two months), peduncles, tepals, stamens, and pistils of *L. formolongi* were harvested for organ-specific expression analysis, immediately frozen in liquid nitrogen, and stored at −80 °C for subsequent organ-specific expression analysis via qPCR.

### 2.3. Molecular Cloning of EST Sequences

The 12 defense-related EST nucleotide sequences retrieved from the NCBI database were designated *L. formolongi* 1–12 (*Lf1–Lf12*) (Table S1). Based on qPCR relative expression levels of 12 ESTs genes under biotic and abiotic stress conditions in *L*. *formolongi* in this study (data not shown), attempts were made to clone all 12 EST sequences, but only seven ESTs were successfully cloned (*Lf6–Lf12*) to obtain full length mRNA sequences (Table S1). For EST sequencing, total RNA was extracted from *Lilium formolongi* ‘Sinnapal lily’ control leaves using an RNeasy mini kit (Qiagen, Hilden, Germany) and treated with RNase-free DNase (Promega, Madison, WI, USA) to synthesize the 3′ ends of cDNA using a 3′ FULL RACE Core Set (Takara, Shiga, Japan) with Oligo dT-3 sites Adaptor Primer (AP) (5′-CTGATCTAGAGGTACCGGATCC-3′) and AMV reverse transcriptase XL. The PCR conditions for cDNA synthesis were the following: 30 °C for 10 min, 50 °C for 30 min, 95 °C for 5 min, and 5 °C for 5 min. To obtain the 3′ sequences of the genes, gene-specific primers were designed and ligated to the Oligo dT 3-sites AP sequence following the manufacturer’s protocol (Table S2). The PCR conditions were as follows: 30 cycles at 94 °C for 30 s, 55 °C for 30 s, and 72 °C for 5 min using Takara LA Taq (Takara, Shiga, Japan). The amplified DNA fragments from RT-PCR were purified using a Gel Extraction kit (Promega, Madison, WI, USA), cloned into the pTOP TA V2 vector, and transformed into competent *Escherichia coli* DH5α cells (Enzynomics, Daejeon, Korea). Plasmid DNA was extracted using a Plasmid Mini kit (Qiagen, Hilden, Germany) and sequenced by Standard-Sequencing (Macrogen, Seoul, Korea). The sequenced RACE products were checked by overlapping with the proper initial cDNA fragments. The sequence data were analyzed using the BLAST program from NCBI. To amplify the 5′ terminal region of cDNA, 5′ RACE PCR was performed based on the 3′ cloned sequences. The cDNA for 5′ RACE PCR was synthesized by RT-PCR of total RNA from control leaf tissue using 5' end-phosphorylated RT primer (Table S3) and a 5′ FULL RACE Core Set (Takara, Shiga, Japan) following the manufacturer’s instructions. Two-step PCR was performed using two sets of gene-specific forward and reverse primers with Takara LA Taq (Takara, Shiga, Japan) at the specified annealing temperature. After the second PCR step, amplified products were ligated into PCR 2.4 vectors (TOPO-TA Cloning kit, Invitrogen, Carlsbad, CA, USA) and sequenced as described above. The sequenced RACE products were combined by overlapping with the initial cloned cDNA fragments, followed by BLAST analysis using nucleotide and deduced protein sequences from NCBI.

### 2.4. Sequence Analysis of mRNA and Deduced Proteins

A protein homology study was carried out for the seven genes successfully cloned from ESTs using BLASTP from the NCBI database [29] (Table S4). The web tool SMART from European Molecular Biology Laboratories (EMBL) [30] was used to identify the different domains in the putative proteins. The primary structures of the genes were analyzed using ProtParam [31], and subcellular protein localization within the cell was determined using ProtComp version 9.0 from SoftBerry [32]. A multiple protein sequence alignment was conducted using Clustal Omega [33,34]. Editing and visualization of the sequence alignment were performed with Jalview, version 2.10.1 [35]. A multiple nucleotide sequence comparison was conducted with ClustalW [36]. Protein-protein interaction using molecular action networks were obtained using STRING software version 10.0 [37].

### 2.5. Phylogenetic Relationship and Motif Analysis

Proteins of *L. formolongi* with the reference proteins including *Arabidopsis* AtHsp70 [38] and AtHsp90 [39] retrieved from NCBI database were aligned by ClustalW program [36] and phylogenetic trees were constructed using the neighbor joining (NJ) algorithm in MEGA 6.06 [40,41]. A bootstrap analysis with 1000 replicates was used to evaluate the significance of the nodes. Pairwise gap deletion mode was considered to confirm that the divergent domains could contribute to the topology of the tree.

Multiple EM for Motif Elicitation (MEME) software (Version 4.11.2) was used to investigate the conserved motifs of the protein sequences [42]. The MEME search setting was developed to acquire the maximum number of motifs (10) with the optimum motif width range (6 to 50).

### 2.6. Biotic Stress Treatments with Botrytis Spp.

Two *Botrytis spp*., *Botrytis cinerea* and *Botrytis elliptica*, were cultured on Petri dishes containing potato dextrose agar (PDA) medium at 20 °C under near-UV light for 10 days until sufficient conidia appeared [7]. The conidia were collected by lightly vortexing in Tween-20 solution (0.05% Tween 20 in sterilized distilled water) [43], and sticky spores were removed from the agar medium with sterilized brushes. Suspended conidia were collected with sterilized pipettes and filtered through four layers of sterile Miracloth (Cat: 475855-1R, Calbiochem^®^, Billerica, MA, USA). The concentration of the conidial suspension was measured with a hemocytometer and adjusted to 5 × 10^4^ conidia/mL with sterile distilled water [43]. Four-month-old *L. formolongi* seedlings were evenly sprayed with *B. cinerea* or *B. elliptica* conidial suspension until the spore solution ran off of the leaf surface. Control and mock plants for both fungi were sprayed separately with pathogen-free distilled water. *Botrytis*-treated and mock-treated plants were transferred into separate polythene protected systems in a growth chamber and provided with mist to maintain 100% relative humidity for the first 48 h. Samples were harvested from infected and mock-treated plants at 0 h, 8 h, 1 d, 2 d, 3 d, 4 d, 5 d, and 7 days post inoculation (dpi) for *B. cinerea* and *B. elliptica* treatments. The youngest 2–3 leaves without midribs were harvested, snap-frozen in liquid nitrogen, and stored at −80 °C until use for RNA extraction. The disease progress index (%) for both fungi was recorded by visual scoring on a time-course basis.

### 2.7. Abiotic Stress Treatments

For abiotic stress treatments, *L. formolongi* seeds were surface disinfected with 70% ethanol for 30 s [44], followed by 1% NaClO plus 0.1% Tween-20 for 10 min in 5 mL Falcon tubes with gentle inversion, and rinsed three times with distilled water [44]. The surface-sterilized seeds were incised with a scalpel at both edges without disturbing the cotyledon to accelerate germination [44]. The sterilized, incised seeds were aseptically grown on fresh liquid half-strength MS medium (MSH) [45] in a culture room under a 16 h light photoperiod at 25 °C. After two months of growth, the seedlings were transferred to freshly prepared liquid MSH and were subjected to four different abiotic stress treatments: cold, heat, drought, and salt. To induce cold and heat stress, the seedlings were incubated at 4 °C and 37 °C for 48 h, respectively [46]. Drought treatment was applied by incubating the seedlings on 150 mm qualitative filter paper (Advantec^®^, Tokyo, Japan) in a culture room under a 16 h light photoperiod at 25 °C for up to 48 h [47]. For salt treatment, the roots of seedlings were soaked in 200 mM NaCl solution at 25 °C for up to 48 h [46]; mock-treated plants were treated with water. The youngest 2–3 leaves and roots were sampled separately at 0 h, 2 h, 8 h, 16 h, 24 h, and 48 h after stress treatments, frozen immediately in liquid nitrogen, and stored at –80 °C for RNA isolation.

### 2.8. RNA Extraction from Various Plant Organs and Stress-Treated (Biotic and Abiotic) Samples

Total RNA was extracted from the roots, stems, leaves, peduncles, tepals, stamens, and pistils of control plants, whereas only leaf samples were collected from mock and stress-treated plants. Total RNA was extracted using an RNeasy mini kit (Qiagen, Hilden, Germany), followed by treatment with RNase-free DNase (Promega, Madison, WI, USA) to remove any traces of genomic DNA contaminants. The cDNA synthesis performed using a Superscript^®^ III First-Strand Synthesis kit (Invitrogen, Carlsbad, CA, USA) according to the manufacturer’s instructions. The relative expression levels among different tissues are compared with the transcript level of roots tissue similar to Khatun et al. [48,49].

### 2.9. Real-Time Quantitative PCR Expression Analysis of Different Organs and Stress-Treated Samples

Gene-specific primers for all seven genes were used for qPCR, whereas the housekeeping *Ll-Actin* primers from *L. longiflorum* (DQ019459) was used as the internal control in all analyses [50]. The qPCR was performed for the seven genes using 1 μL cDNA from all seven tissues including roots, stems, leaves, peduncles, tepals, stamens, and pistils of control; and mock and stress-treated *L. formolongi* separately in a 20 μL reaction volume containing 2× qPCR BIO SyGreen Mix Lo-Rox SYBR^®^ Green Super-mix with ROX (PCR Biosystems Ltd., London, UK). The qPCR conditions were as follows: pre-incubation at 95 °C for 10 min, followed by three-step amplifications at 95 °C for 20 s, 58 °C for 20 s, and 72 °C for 25 s for 40 cycles. The melting conditions were 95 °C for 10 s, 65 °C for 60 s, and 97 °C for 1 s as a default setting. For quantification, the fluorescence was recorded following the last step of each cycle, and three replicates (*n = 3*) were averaged per sample. Amplification, detection, and data analysis were carried out using a LightCycler96 system (Roche, Mannheim, Germany). The relative gene expression levels were calculated using the 2^−∆∆ct^ method [51].

### 2.10. Statistical Analyses

Gene expression levels obtained by qPCR were analyzed using a general linear model (GLM) with a completely randomized design (CRD) for analysis of variance (ANOVA). A Tukey test was conducted to identify differences between and among treatments and sampling points. A *p*-value less than 0.05 was considered to indicate statistical significance. All analyses were carried out using Statistical Analysis System (SAS) version 9.1 [52].

## 3. Results

### 3.1. Sequence Analysis of Defense-Related ESTs from L. formolongi

We retrieved 12 defense related ESTs from NCBI and designated them as *L. formolongi* 1–12 (*Lf1–Lf12*) (Table S1). NCBI nucleotide BLAST analysis showed that the putative stress-related proteins share high sequence similarity with defense-related proteins from various plant species (Table S1) [53,54,55,56,57,58,59,60,61]. The sequence similarity of ESTs with homologs from other plant species ranged from 75 to 85% (Table S1).

### 3.2. Molecular Cloning and Sequence Analysis of Stress-Related Genes

We successfully cloned and obtained the full-length mRNA sequence of seven EST genes using RACE PCR and submitted them to the NCBI under the GenBank accession numbers of KX683995–KX684001 (Table 1 and Figure S1). NCBI BLAST analysis of the deduced protein sequences including *LfHsp70-1*, *LfHsp70-2*, and *LfHsp70-3* share 92% to 94% similarity with HSP70 reference proteins from other species (Table S4) [57,62,63,64,65,66,67,68,69,70,71,72,73]. Protein domain organization and multiple alignment with the reference proteins showed that all three *LfHsp70* proteins contain a highly conserved HSP70 domain (heat shock family protein 70) (Table 1 and Figure S2). Therefore, we renamed these three proteins *L. formolongi* heat shock protein 70-1 (*LfHsp70-1*), *LfHsp70-2*, and *LfHsp70-3* (Table 1). The fourth protein, LfHsp90 (Lf9 EST), shares 90–93% similarity with HSP90 reference proteins (Table S4) and contains a highly conserved HATPase, and an HSP90 domains (heat shock family protein 90) (Table 1 and Figure S3), so we renamed this protein *L. formolongi* heat shock family protein 90 (LfHsp90). The fifth protein on the list, LfUb, shares 62–73% similarity with the reference ubiquitin domain containing proteins (Table S4) and contains three characteristic conserved domains namely an ubiquitin homolog domain (UBQ), two ubiquitin-associated domains (UBA), and stress-inducible phosphoprotein motif (STI1) (Table 1 and Figure S4) and thus, we renamed this protein *L. formolongi* ubiquitin domain containing protein (LfUb). The sixth protein, *LfHsp70* protein, shares 63–73% similarity with the reference cytochrome-b5 domain containing proteins (Table S4). Protein domain organization and multiple alignment revealed that *LfHsp70* contains a conserved transmembrane region and a cytochrome-b5 heme/steroid binding domain (Table 1 and Figure S5) and hence, we renamed this protein *L. formolongi* cytochrome-b5 steroid binding domain containing protein (LfCytb5). Finally, the seventh protein, LfRab, shares 92–95% similarity with the reference proteins (Table S4) and contains a conserved RAB domain (Table 1 and Figure S6) and thus, we renamed this protein *L. formolongi* Rab domain containing protein (LfRab). The predicted molecular weights (MW) of the deduced proteins ranged from 22.86 to 80.23 kD, and their isoelectric points (pIs) ranged from 4.55 to 6.51 (Table 1). *LfHsp70-1*, *LfHsp70-2*, and *LfHsp70-3* were predicted to be localized to the cytoplasm and nucleus, whereas LfHsp90 is seemed to be localized to the cytoplasm and membranes (Table 1). LfUb appeared to be localized in both cytoplasm and nucleus (Table 1), whereas LfCytb5 is expected to be localized to the plasma membrane, and LfRab is seemed to be localized to the cytoplasm, Golgi, and endoplasmic reticulum (Table 1).

### 3.3. Phylogenetic Relatedness and Motif Distribution of Defense-Related Proteins

**Figure 1:**
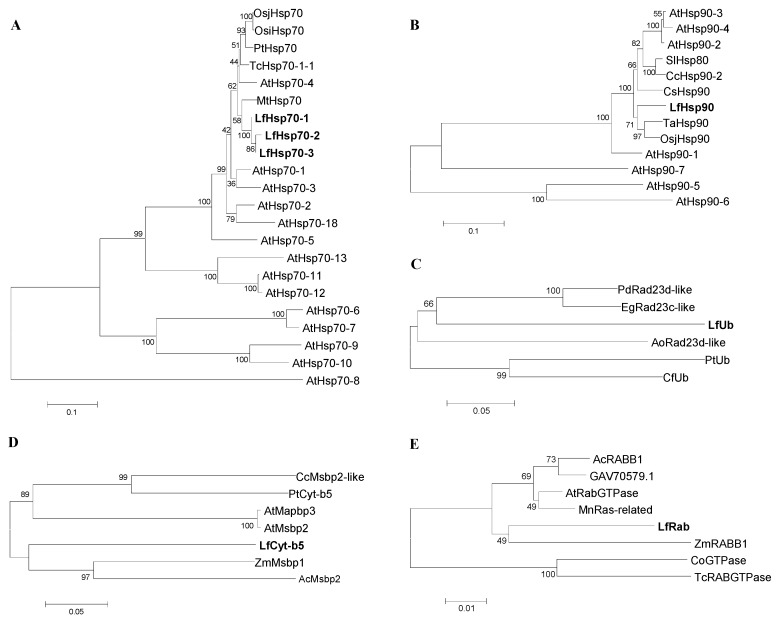
Phylogenetic analysis of seven putative stress-related *Lilium formolongi* proteins (highlighted in bold font; (**A**) Hsp70 family protein, (**B**) Hsp90 family protein, (**C**) Ubiquitin domain containing protein, (**D**) Cytochrome-b5 domain containing protein, and (**E**) RAB domain containing protein) with the respective reference proteins from other plant species. The phylogenetic trees were constructed with MEGA 6.06 using the neighbor-joining method. The results were confirmed using bootstrap analysis, with support values at the nodes representing percentages from 1000 repetitions. The scale represents the frequency of amino acid substitutions between sequences, as determined by the Poisson evolutionary distance method.

The phylogenetic relatedness showed that the three putative proteins LfHsp70-1, LfHsp70-2, and LfHsp70-3; and LfHsp90 were distributed with the cytosolic reference proteins with strong bootstrap support (Figure 1A,B and Table S4) [38,39]. Stress-related protein LfUb was related with the ubiquitin domain containing protein (Figure 1C and Table S4); LfCytb5 was related with the cytochrome b5 domain containing protein (Figure 1D and Table S4); and LfRab was related with the Rab domain containing protein (Figure 1E and Table S4), with strong bootstrap support. GenBank accession numbers of the abbreviated reference proteins used to construct the trees are listed in Table S4.

Analysis of motif distribution compared with reference proteins showed that LfHsp70*-1* and *LfHsp70-3* contain nine identical conserved motifs, whereas LfHsp70*-2* contains five identical conserved motifs, each containing 29–50 amino acids (Figure S7A) and LfHsp90 has 10 conserved motifs, each containing 50 amino acids (Figure S7B). LfUb contains 10 motifs ranging from 11–50 amino acids long (Figure S7C), whereas LfHsp70 has five conserved motifs ranging in size from 6–50 amino acids (Figure S7D). Finally, LfRab has six out of 10 conserved motifs, each comprising 11–50 amino acids (Figure S7E).

### 3.4. Organ-Specific Expression Analysis of Defense-Related Genes

**Figure 2:**
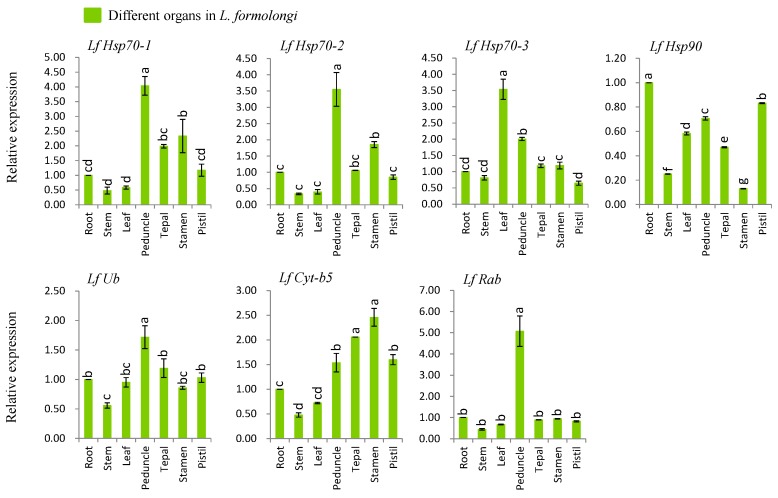
Real-time quantitative PCR analysis of seven stress-related genes in different *L. formolongi* tissues. The error bars represent the standard error of the means of three independent replicates. Different letters (a, b, c, d, e, f, g) obtained from Tukey’s pairwise comparison test indicate statistically significant differences (*p* < 0.05) in relative expression of each gene at different time-points after treatment.

We investigated the organ-specific expression patterns of seven genes viz., *LfHsp70-1*, *LfHsp70-2*, *LfHsp70-3*, *LfHsp90*, *LfUb*, *LfCyt-b5*, and *LfRab* using different tissues including roots, stems, leaves, peduncles, tepals, stamens, and pistils of *L. formolongi* line ‘Sinnapal lily’ by qPCR (Figure 2). The maximum expression (up-regulated between 1.7- and 5-fold) were observed at peduncle tissue for *LfHsp70-1*, *LfHsp70-2*, *LfUb*, and *LfRab* (Figure 2). The *LfHsp70-3* showed the maximum expression (up-regulated 3.5-fold) in leaf whereas the *LfCyt-b5* showed the highest expression (up-regulated 2.5-fold) in stamen (Figure 2). The *LfHsp90* displayed the down-regulation in all tissues giving the minimum expression (down-regulated 6-fold) in stamen (Figure 2).

### 3.5. B. elliptica Is More Virulent than B. cinerea

**Figure 3:**
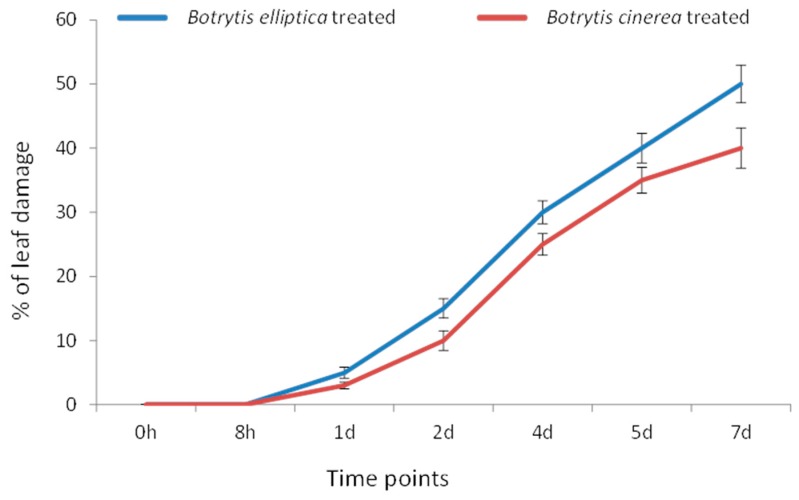
*Botrytis elliptica*- and *Botrytis cinerea*-inoculated susceptible *L. formolongi* line Sinnapal lily showing disease progression at different time points.

Water-soaked lesions first formed at one day post inoculation (dpi) with both *B. elliptica* and *B. cinerea*. However, the leaf area occupied by lesions was greater under *B. elliptica* infection (55%) than under *B. cinerea* infection (45%) at 7 dpi (*p* < 0.05) (Figure 3).

### 3.6. qPCR Expression Analysis after Biotic Stress

#### 3.6.1. Gene Expression in *B. cinerea*-Inoculated Susceptible *L*. *formolongi*

**Figure 4:**
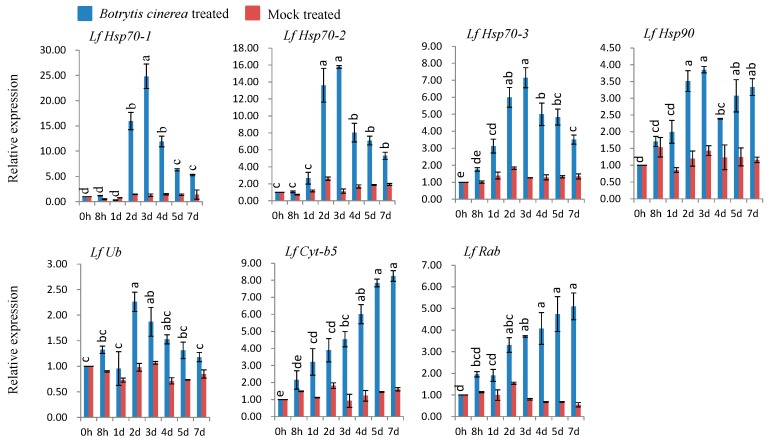
Real-time quantitative PCR to investigate the relative expression of seven stress-induced genes in *L. formolongi* line Sinnapal lily infected by *B. cinerea*. The error bars represent the standard error of the means of three independent replicates. Different letters (a, b, c, d, e) obtained from Tukey’s pairwise comparison test indicate statistically significant differences (*p* < 0.05) in relative expression of each stress-induced gene at different time-points after treatment.

All seven genes (*LfHsp70-1*, *LfHsp70-2*, *LfHsp70-3*, *LfHsp90*, *LfUb*, *LfCyt-b5*, and *LfRab*) were markedly up-regulated upon *B. cinerea* inoculation compared to both control and mock-treated samples (Figure 4). The maximum expression levels (up-regulated between 3.8- and 25-fold versus the control) were observed at three days post inoculation for *LfHsp70-*1, *LfHsp70-2*, *LfHsp70-3*, and *LfHsp90* (Figure 4). Two other genes, *LfCyt-b5* and *LfRab*, displayed the highest expression levels at 7 dpi (up-regulated between 8.2- and 5-fold compared to the control; Figure 4). *LfUb* exhibited the highest expression level at 3 dpi (up-regulated 2.3-fold compared to the control; Figure 4).

#### 3.6.2. Gene Expression in *B*. *elliptica*-Inoculated Susceptible *L*. *formolongi*

**Figure 5:**
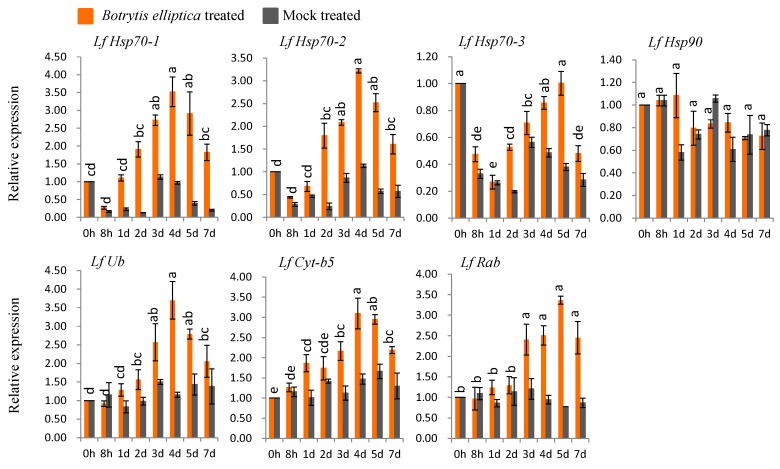
Real-time quantitative PCR to investigate the relative expression of stress-induced genes in susceptible *L. formolongi* line Sinnapal lily infected by *B. elliptica*. The error bars represent the standard error of the means of three independent replicates. Different letters (a, b, c, d, e) obtained from Tukey’s pairwise comparison test indicate statistically significant difference (*p* < 0.05) in relative expression of each stress-induced gene at different time-points after treatment.

Five genes (*LfHsp70-1*, *LfHsp70-2*, *LfUb*, *LfCyt-b5*, and *LfRab*) were up-regulated under *B. elliptica* inoculation compared to both control and mock-treated samples (Figure 5). The highest expression levels (3- to 3.2-fold versus the control) were observed at 4 dpi for *LfHsp70-*1 and *LfHsp70-2*, respectively compared to the control (Figure 5). *LfUb* was up-regulated 3.7-fold at 4 dpi, whereas *LfCyt-b5* and *LfRab* showed the highest levels of up regulation (3.1- and 3.4-fold, respectively versus the control) at 5 dpi (Figure 5). By contrast, *LfHsp70-3* and *LfHsp90* were down-regulated at almost all time points compared to the control (Figure 5).

### 3.7. Gene Expression under Abiotic Stress Conditions

#### 3.7.1. Gene Expression under Heat Stress

**Figure 6:**
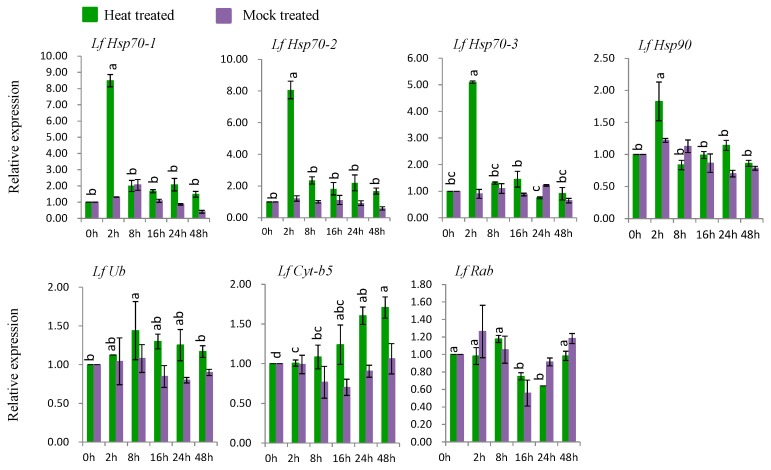
Real-time quantitative PCR to investigate the relative expression of stress-induced genes in response to heat stress treatments in *L. formolongi* line Sinnapal lily. The error bars represent the standard error of the means of three independent replicates. Different letters (a, b, c, d) obtained from Tukey’s pairwise comparison test indicate statistically significant differences (*p* < 0.05) in relative expression of each stress-induced gene at different time-points after treatment.

Six genes (*LfHsp70-1*, *LfHsp70-2*, *LfHsp70-3*, *LfHsp90*, *LfUb*, and *LfCyt-b5*) were up-regulated at various time points after heat-stress treatment compared to both control and mock-treated samples (Figure 6). *LfHsp70-1*, *LfHsp70-2*, *LfHsp70-3*, and *LfHsp90* were up regulated 8.5-, 8.0-, 5-, and 1.8-fold, respectively, at 2 h after heat-stress treatment compared to the control (Figure 6). *LfUb* and *LfCyt-b5* were up-regulated 1.4-fold at 8 h after heat-stress treatment and 1.7-fold at 48 h after stress treatment, respectively, compared to the control (Figure 6). Finally, *LfRab* was down regulated at 16 and 24 h after heat-stress treatment compared to the control (Figure 6).

#### 3.7.2. Gene Expression under Cold Stress

**Figure 7:**
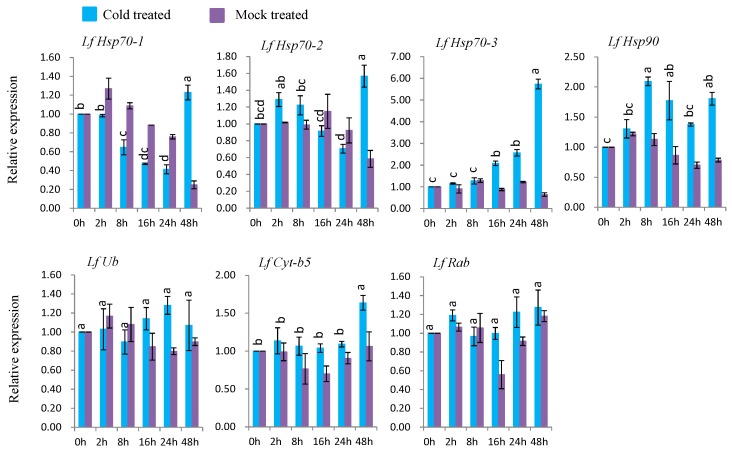
Real-time quantitative PCR to investigate the relative expression of stress-induced genes in response to cold stress treatments in *L. formolongi* line Sinnapal lily. The error bars represent the standard error of the means of three independent replicates. Different letters (a, b, c, d) obtained from Tukey’s pairwise comparison test indicate statistically significant differences (*p* < 0.05) in relative expression of each stress-induced gene at different time-points after treatment.

Under cold treatment, five genes (*LfHsp70-1*, *LfHsp70-2*, *LfHsp70-3*, *LfHsp90*, and *LfCyt-b5*) were up-regulated at different time points after cold-stress treatment compared to both control and mock-treated samples (Figure 7). *LfHsp70-1*, *LfHsp70-2*, *LfHsp70-3*, *LfHsp90*, and *LfCyt-b5* were up-regulated (1.4-, 1.5-, 5.8-, 2.1-, and 1.8-fold, respectively) at 48 h after cold-stress treatment compared to the control (Figure 7). The two remaining genes, *LfUb* and *LfRab*, were not induced in cold stress-treated plants compared to the control (Figure 7).

#### 3.7.3. Gene Expression under Drought Stress

**Figure 8:**
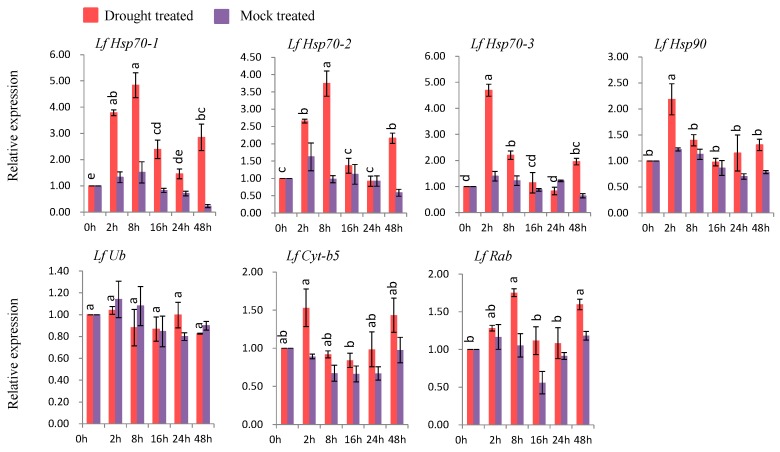
Real-time quantitative PCR to investigate the relative expression of stress-induced genes in response to drought stress treatments in *L. formolongi* line Sinnapal lily. The error bars represent the standard error of the means of three independent replicates. Different letters (a, b, c, d, e) obtained from Tukey’s pairwise comparison test indicate statistically significant differences (*p* < 0.05) in relative expression of each stress-induced gene at different time-points after treatment.

Six genes (*LfHsp70-1*, *LfHsp70-2*, *LfHsp70-3*, *LfHsp90*, *LfCyt-b5*, and *LfRab* were up-regulated at different time points after drought-stress treatment compared to both control and mock-treated samples (Figure 8). *LfHsp70-1*, *LfHsp70-2*, and *LfRab* were up-regulated 4.9-, 3.7-, and 1.7-fold, respectively, at 8 h after drought-stress treatment compared to the control (Figure 8). *LfHsp70-3* and *LfCyt-b5* showed the highest levels of upregulation (4.8- and 1.5-fold, respectively, compared to the control) at 2 h after drought-stress treatment (Figure 8). By contrast, *LfUb* was not up-regulated in drought stress-treated plants compared to the control (Figure 8).

#### 3.7.4. Gene Expression under Salt Stress

**Figure 9:**
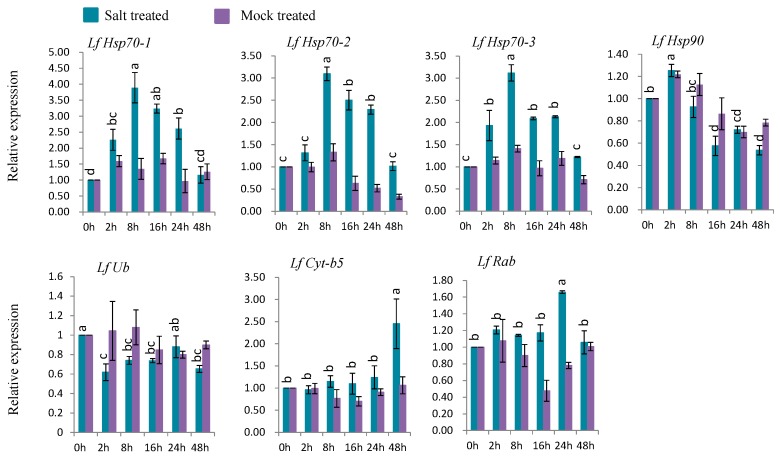
Real-time quantitative PCR to investigate the relative expression of stress-induced genes in response to salt stress treatments in *L. formolongi* line Sinnapal lily. The error bars represent the standard error of the means of three independent replicates. Different letters (a, b, c, d) obtained from Tukey’s pairwise comparison test indicate statistically significant differences (*p* < 0.05) in relative expression of each stress-induced gene at different time-points after treatment.

Five genes (*LfHsp70-1*, *LfHsp70-2*, *LfHsp70-3*, *LfCyt-b5*, and *LfRab*) were up-regulated at different time points after salt-stress treatment compared to both control and mock-treated samples (Figure 9). *LfHsp70-1*, *LfHsp70-2*, and *LfHsp70-3* were highly expressed (up-regulated 3.9-, 3.1-, and 3.1-fold, respectively) at 8 h after salt-stress treatment compared to the control (Figure 9). *LfRab* and *LfCyt-b5* exhibited the highest levels of upregulation (1.6- and 2.5-fold) at 2 h after stress treatment, respectively, compared to the control (Figure 9). *LfHsp90* was up regulated only at 2 h after salt-stress treatment, followed by down regulation, while *LfUb* was down-regulated in salt stress-treated plants compared to the control at all time points (Figure 9).

### 3.8. Analysis of Stress-Related Protein Interactions

**Figure 10:**
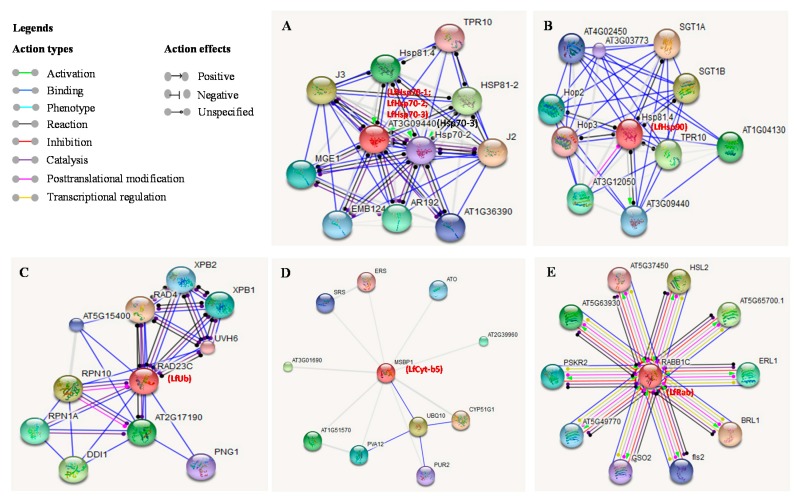
Interaction network of the seven stress-related proteins identified in *L. formolongi* with related proteins in *Arabidopsis* (**A**–**E**). Action types and action effects of predicted functional partners are indicated by different-colored lines.

We investigated the physical and molecular action networks of the seven stress-related *L. formolongi* proteins and their association with the top-10 *Arabidopsis* proteins (Figure 10). *LfHsp70-1*, *LfHsp70-2*, and *LfHsp70-3* are highly homologous to *Arabidopsis* Hsp70-3 (AT3G09440), which stabilizes pre-existent proteins against aggregation and mediates the folding of newly translated polypeptides in the cytosol and within organelles [74]. The *Arabidopsis* Hsp70-3 homolog closely interacts with the Hsp70-2 isoform, indicating that these isoforms are functionally similar (Figure 10A). Therefore, different isoforms of *LfHsp70* might interact and function as co-activators during plant growth, development, and stress defense responses (Figure 10A) [74]. LfHsp90 is highly homologous to *Arabidopsis* Hsp81.4, which associates with proteins such as hormone receptors and some classes of kinases and is implicated in signal transduction and development (Figure 10B) [75]. Again, *Arabidopsis* Hsp81.4 positively interacts with Hsp70-3 (AT3G09440) during various developmental phases in cells [75]. Similarly, LfHsp90 might play an important role in plant signal transduction [39]. LfUb is highly homologous to RAD23C (RADIATION SENSITIVE 23C), which might be involved in nucleotide excision repair (Figure 10C). RAD23C also interacts with RPN1A, which is required during embryogenesis [76] and for optimal plant growth and stress responses [77]. Hence, LfUb might interact with different functional partners in various growth, development, and stress-response mechanisms. LfCy-b5 is highly homologous to MSBP1 (membrane steroid binding protein 1) that modulates cell elongation and brassinosteroid signaling and may function as a co-receptor with BAK1, resulting in increased endocytosis [78]. This protein also interacts with CYP51G1 (cytochrome P450 51G1) by binding with UBQ10 (polyubiquitin 10), which is induced by salicylic acid (Figure 10D), suggesting that it might be involved in providing defense against stress. LfRab is highly homologous to RABB1C (RAB GTPase homolog B1C), which functions in intracellular vesicle trafficking and protein transport (Figure 10E). Again, RABB1C binds to fls2 (FLAGELLIN-SENSITIVE 2), comprising the pattern-recognition receptor (PPR), which recognizes flagellin (flg22), a potent elicitor of the defense response, leading to pathogen-associated molecular pattern (PAMP)-triggered immunity.

## 4. Discussion

Based on stress-induced expression profiling of 12 defense-related EST genes under stress conditions in this study (data not shown), we successfully cloned seven defense-related candidate genes from *L. formolongi* line Sinnapal (Table 1). We then obtained seven complete mRNAs corresponding to the seven selected EST genes via RACE-PCR. Seven genes and their deduced protein sequences were analyzed in silico. We conducted a relative expression analysis by qPCR of these seven genes in various organ tissues of control plants; and under *B. cinerea*, *B. elliptica* infection, and four abiotic stress including heat-, cold-, drought-, and salt-treated leaf tissues to explore the stress-responsive expression patterns of these genes in *L. formolongi*.

### 4.1. Importance of Sequence Variation in Defense-Related Proteins

Protein sequence alignment revealed that all three *LfHsp70* isoforms, namely *LfHsp70-1*, *LfHsp70-2*, and *LfHsp70-3*, contain highly conserved, N-terminal ATPase domains [79] and C-terminal peptide-binding domains [80] followed by a substrate-binding consensus nuclear localization signal ‘GTPIEEVD’ (Figure S2) [38]. The intrinsic ATPase activity of Hsp70 is required for it to form hetero-complexes with co-chaperones such as DnaJ/Hsp40 and GrpE and is involved in the successive cycles of substrate binding and release [81]. Therefore, hetero-complex activity might vary due to the various deletions of N-terminal amino acids in *LfHsp70-1*, *LfHsp70-2*, and *LfHsp70-3* (Figure S2). Moreover, higher protein similarity of these proteins suggests that they might be functionally redundant [56]. At least three cytoplasmic Hsp70s exist in plants [82]. Phylogenic analysis indicated that similar to Hsp70 family members of *Arabidopsis*, AtHsp70 (AtHsp70-1 to AtHsp70-5; AtHsp70-18), lily Hsp70 genes *LfHsp70-1*, *LfHsp70-2*, and *LfHsp70-3* could be localized in cytoplasm (Figure 1A) [38]. Similar to Hsp90 of *Arabidopsis* AtHsp90 (AtHsp90-1 to AtHsp90-4), LfHsp90 could also be localized in cytoplasm (Figure 1B) [39,83]. LfHsp90 contains a highly conserved N-terminal ATP binding domain attached to a highly conserved C-terminal region by a “charged linker”, which varies in length and composition among species and isoforms (Figure S3) [84]. Importantly, conformational changes occur in Hsp90s due to the intrinsic ATPase activity (Figure S3) [85]. LfHsp90 contains a leucine zipper motif in the middle of the polypeptide shared with the five reference members (Figure S3), which might contribute to its specificity and stability during the dimerization process with several transcription factors [86]. Sequence alignment showed that LfUb contains a highly conserved UBQ domain (Figure S4) [87], which might interact with other proteins through its post-translational attachment (ubiquitination) and modify their functions, locations, or trafficking patterns [88]. Two other UBA domains are separated by STI1, and N- and C-termini of STI1 can bind to Hsp70 and Hsp90, respectively, which might facilitate the trafficking of a variety of proteins through the cytoplasm Figure S4 [89] or may function as chaperone complexes [90]. Plasma membrane-localized *LfHsp70* protein includes cytochrome-b5 heme/steroid binding domains found in a diverse range of proteins (Figure S5). However, LfRab along with other Rab domain containing proteins vary mostly at their carboxyl termini, which function in subcellular targeting due to the well-conserved guanine-nucleotide binding region (Figure S6) [22].

### 4.2. L. formolongi Defense-Related Genes Are Active in All Organs

We investigated the expression patterns of *LfHsp70-1*, *LfHsp70-2*, *LfHsp70-3*, *LfHsp90*, *LfUb*, *LfHsp70*, and *LfRab* in various organs including roots, stems, leaves, peduncles, tepals, stamens, and pistils (Figure 2). Differential expression patterns of *LfHsp70-1*, *LfHsp70-2* and *LfHsp70-3* in different tissues indicate that various isoforms of *LfHsp70* genes are expressed constitutively and are functionally redundant. Accumulating evidence shows that various Hsp70 family members and *LfHsp90* play constitutive but functionally distinct roles in growth and development in different plants [74,82,91,92]. Differential upregulated expression of three other family genes, *LfUb*, *LfCyt-b5*, and *LfRab*, in all organs suggests that they might be involved in growth and developmental processes in lily (Figure 2). Reports revealed that Rab family genes are up-regulated during growth and development in legumes [93].

### 4.3. Expression Analysis of L. formolongi Defense-Related Genes under Botrytis Infection

Since plants are continuously challenged by a variety of biotic and abiotic stresses, they have evolved many stress-tolerance and defense mechanisms to reduce damage [94]. *Botrytis spp.* secretes an elicitor that triggers a series of rapid host responses in lily, including the production of transient Ca^+2^ ion fluxes, followed by the production of reactive oxygen species (ROS), resulting in plant death [95,96]. Conversely, ROS can function as a cross-linking agent during the hypersensitive response (HR), as well as activating cytosolic Ca^2+^ signaling cascades to induce R-gene-mediated disease-resistance mechanisms [96]. In *Arabidopsis*, an immune response is regulated by cytosolic Hsp70, together with SGT1 (suppressor of G2 allele kinetochore protein), under pathogenic stress conditions [97] and is involved in the stability of R proteins, cell death, and the positive regulation of immunity [98,99]. Hsp90, together with SGT1 and RAR1 (required for *Mla12* resistance), likely regulate the activity and stability of R proteins, which accumulate due to the recognition of pathogen-derived effectors, resulting R-mediated disease resistance in *Arabidopsis* [100,101]. In addition, silencing of *Hsp90* reduces the accumulation of R proteins in tomato, confirming its role in R-mediated disease resistance [99]. In the current study, *LfHsp70-1*, *LfHsp70-2*, *LfHsp70-3*, and LfHsp90 were strongly induced by *B. cinerea*, whereas *LfHsp70-1* and *LfHsp70-2* were specifically induced by *B. elliptica*, suggesting that all three members of the cytosolic *LfHsp70* family and the cytosolic LfHsp90 protein are not equally active and do not regulate the accumulation of R proteins during the R-gene mediated disease resistance response under various pathogen stresses, specifically *Botrytis spp* (Figure 4 and Figure 5)*. LfUb* was more responsive to *B. elliptica* than to *B. cinerea* at later time points (Figure 4 and Figure 5). Since a glucan fungal elicitor secreted by *Phytophthora megasperma* up- regulates ubiquitin transcripts in soybean cells [99], we speculate that effectors secreted by *B. elliptica* might increases *LfUb* transcript levels more strongly than those of *B. cinerea*, resulting in a stronger resistance interaction. Two other genes, *LfCyt-b5* and *LfRab*, were more responsive to *B. cinerea* than to *B. elliptica* (Figure 4 and Figure 5). Cytochrome-b5 increases plant adaptation to stress by maintaining membrane fluidity through the desaturation of fatty acids under adverse environmental conditions [21]. Furthermore, cytochrome-b5, along with cytochrome P450 enzymes, are thought to be a source of ROS [20,102] under biotic stress conditions, which might contribute to disease tolerance in lily [103,104,105]. However, in *Arabidopsis AtRabG3e* (*Rab7*) is induced during biotic stresses due to infection by *B. cinerea*, leading to hypersensitive cell death [106,107]. AtRabGTPases are involved in intracellular vesicle trafficking, resulting in plant adaptation to pathogen stress [23,107].

### 4.4. Expression Analysis of L. formolongi Defense-Related Genes under Heat Stress

Six genes, *LfHsp70-1*, *LfHsp70-2*, *LfHsp70-3*, *LfHsp90*, *LfUb*, and *LfCyt-b5*, were significantly induced under heat treatment (Figure 6). In this study, the cytosolic chaperone gene *LfHsp90* was expressed at much lower levels compared to the other *LfHsp70* genes under heat-stress conditions during the same stress periods (Figure 6), suggesting that *LfHsp70* is more responsive to heat stress than *LfHsp90* in lily [108]. Importantly, the up-regulation of *Hsp70* increases tolerance to endogenous oxidative damage under heat-stress conditions in transgenic chrysanthemum [105] and *Arabidopsis* [109]. Although the exact mechanism remains to be determined, Hsps might transmit heat shock signals through the Ca^2+^-CaM (calmodulin) pathway in lily; wheat plants under heat-stress conditions generate high levels of cytoplasmic Ca^2+^, which activates CaM production, subsequently stimulating the DNA-binding activity of heat-shock factor (HSF). The DNA-binding activity of HSF initiates the transcription and translation of *Hsp* genes, which regulates cellular homeostasis and plant tolerance to heat-stress [17,110]. *LfUb* was also induced by heat-stress conditions at 8 h after treatment (Figure 6). The ubiquitination capacity of plants increases under heat-stress conditions, which mediates plant defense mechanisms [18,19]. In addition, the heat shock chaperonin-binding motif STI1 of LfUb is a key component of Hsp70 or Hsp90 immune hetero-complexes, which regulate plant defense mechanisms [90]. Conversely, *LfCyt-b5* was significantly induced at 48 h after stress treatment, suggesting that it might function in the heat stress response at later stages of treatment (Figure 6).

### 4.5. Expression Analysis of L. formolongi Defense-Related Genes under Cold Stress

Five genes, *LfHsp70-1*, *LfHsp70-2*, *LfHsp70-3*, *LfHsp90*, and *LfCyt-b5*, exhibited variable levels of induction under cold-stress conditions (Figure 7). *LfHsp70-3* was the most highly induced, followed by *LfHsp70-2*, whereas almost no induction was observed for *LfHsp70-1* at 48 h after cold stress treatment, indicating that *LfHsp70* family genes are stimulated to varying degrees at later stages of cold stress compared to the early stages of heat stress (Figure 6 and Figure 7). These data also suggest that all *LfHsp70* members are not equally responsive to cold-stress conditions. Indeed, some but not all *Hsp70* genes are induced under cold stress in *Arabidopsis* [74], spinach, and tomato [82,108]. Conversely, *LfHsp90* was significantly induced at 8 h after treatment compared to the two *LfHsp70* members (Figure 7). Cytosolic *Hsp90* in *Brassica napus* [111] and rice [112] are also induced by low temperature and like these genes, *LfHsp90* might be essential for cold tolerance in lily. Although *LfCyt-b5* remained almost inactive up to 24 h after stress treatment, this gene was significantly induced at 48 h after stress treatment, suggesting that it might functions in cold tolerance mechanisms at later stages in lily (Figure 7).

### 4.6. Expression Analysis of L. formolongi Defense-Related Genes under Drought Stress

Six genes, namely *LfHsp70-1*, *LfHsp70-2*, *LfHsp70-3*, *LfHsp90*, *LfCytb5*-like, and *LfRab*, were significantly induced under drought treatment (Figure 8). Importantly, all three *LfHsp70* genes were induced to varying degrees, but their expression levels were higher than that of *LfHsp90*, indicating that all *LfHsp70* genes play a more active role in drought tolerance than *LfHsp90* (Figure 8). Increasing *Hsp70* expressing under drought stress enhances drought resistance in transgenic tobacco [113] and chrysanthemum [105] by preventing endogenous oxidative stress. Based on their molecular chaperone and regulatory functions, the drought stress response might be mediated by two different Hsp mechanisms [113]. *LfCyt-b5* was significantly induced at 2 h after stress treatment, suggesting that it might function under drought stress at early stages in lily. The upregulation of *LfRab* similar to Rab family gene *AtRabG3e* (*Rab7*) in transgenic *Arabidopsis* plants under drought conditions, suggests that this family gene might be involved in drought-stress tolerance [107].

### 4.7. Expression Analysis Defense-Related of L. formolongi Genes Under Salt Stress

Six genes, *LfHsp70-1*, *LfHsp70-2*, *LfHsp70-3*, *LfHsp90*, *LfCyt-b5*, and *LfRab*, were significantly induced under salt treatment (Figure 9). All three *LfHsp70* genes, *LfHsp70-1*, *LfHsp70-2*, and *LfHsp70-3*, were significantly induced at 8 h after salt-stress treatment, which indicates that they are responsive at an early stage of stress but are not equally involved in the salt tolerance response (Figure 9). The up-regulation of *Hsp70* increases tolerance against endogenous oxidative damage under salt-stress conditions in transgenic chrysanthemum [105] and transgenic tobacco [114]. Again, *LfHsp90* was induced at 2 h after salt-stress treatment, but the fold-change in expression was quite low compared to the *LfHsp70* genes under salt treatment (Figure 9). However, this gene was down-regulated at the end of the stress period, indicating that it may become active at the early stage of the post-stress period but may become inactive at the later stage (Figure 9). The significant up regulation of *LfCyt-b5* at 48 h indicates its responsiveness to salt-stress condition at the later stage of the treatment period (Figure 9). *LfRab* was also significantly induced at 24 h after salt-stress treatment (Figure 9), indicating its possible association with the salt tolerance mechanism. *Arabidopsis* *AtRabG3e* (*Rab7*) [107] and rice *OsRab7* [23] are highly induced under salt-stress conditions, suggesting that this gene family functions in salt tolerance. Indeed, overexpressing these genes increases salt tolerance in plants by enhanced vesicle trafficking.

## 5. Conclusions

Of the 12 defense-related ESTs genes in *L. formolongi* (Easter lily) identified from published data, we successfully cloned seven candidate ESTs by RACE-PCR and obtained their complete mRNA sequences. Sequence alignment of the seven deduced proteins with the reference proteins showed that the respective characteristic domains are well-conserved among proteins. Protein-protein interaction analysis showed that all seven proteins interact with different stress-related proteins, suggesting that they are stress-responsive. The qPCR analysis revealed that these defense-related genes are differentially expressed in all organs, suggesting that they are involved in growth and development. All seven genes were induced by *B. cinerea* inoculation and five genes were induced by *B. elliptica* inoculation, indicating their possible association with disease resistance mechanisms against the respective *Botrytis spp*. Six genes were induced by heat-stress treatment, five were induced by cold-stress treatment, and six were induced by drought and salt stress, suggesting they play multiple roles in stress responses in *L. formolongi*. The seven stress-induced lily genes seem to have a possible involvement in stress-related mechanisms, however they need to be studied further to see if their characterization proves any direct involvement in the increased resistance to biotic and abiotic stress and thus could be candidates to be used in genetic improvement.

## Figures and Tables

**Table 1:** Subcellular localization and structural features of seven stress-related genes in *Lilium formolongi.*

EST Gene Name	Gene Name (mRNA)	GenBank Accession	Location	Domain Name	*E*-Value	Domain Position		CDS (bp)	Protein			Retrieved Sequence
						Start	End		Length (aa)	MW (kDa)	P^I^	
*Lf6*	*LfHsp70-1*	KX683998	Cytoplasm and Nucleus	HSP70	1.2 × 10^−217^	1	513	1623	540	59.51	5.04	1821
*Lf7*	*LfHsp70-2*	KX683999	Cytoplasm and Nucleus	HSP70	3.2 × 10^−113^	1	306	1002	333	36.58	4.78	1238
*Lf8*	*LfHsp70-3*	KX684000	Cytoplasm and Nucleus	HSP70	2.6 × 10^−209^	1	493	1563	520	57.27	5.17	1752
*Lf9*	*LfHsp90*	KX684001	Cytoplasm and Membrane	HATPase_c HSP90	2.81 × 10^−8^ 6.3 × 10^−236^	28 185	183 700	2103	700	80.23	4.90	2422
*Lf10*	*LfUb*	KX683995	Cytoplasm and Nucleus	UBQ UBA STI1 UBA	2.3 × 10^−18^ 8.0 × 10^−5^ 4.81 × 10^−6^ 1.38 × 10^−8^	1 155 253 336	75 195 296 373	1143	380	40.36	4.55	1505
*Lf11*	*LfCyt-b5*	KX683996	Plasma membrane	TM Cyt-b5	- 2.68 × 10^−20^	13 71	35 167	795	264	28.65	4.56	1144
*Lf12*	*LfRab*	KX683997	Cytoplasm and Golgi and Endoplasmic Reticulum	RAB	2.63 × 10^−105^	7	170	627	208	22.86	6.51	950

Abbreviations: EST, expressed sequence tag; CDS, coding DNA sequence; bp, base pair; aa, amino acid; kDa, kilo Dalton; P^I^, iso-electric point; MW, molecular weight; TM, transmembrane.

## Supplementary Materials

The following are available online at www.mdpi.com/2073-4425/8/7/172/s1. Table S1: Nucleotide homology analysis of 12 EST genes from *Lilium formolongi.* Table S2: Specific ligated primer sequences used for 3´ RACE PCR amplification of seven stress-related genes from *L. formolongi*. Table S3: Specific primer sequences used for cDNA synthesis and 5´ RACE PCR amplification of seven stress-related genes from *L. formolongi*. Table S4: Protein homology analysis of stress-related genes in *L. formolongi.* Table S5: Nucleotide sequence relatedness among the seven stress-related genes from *L. formolongi*. Table S6. Specific primer sequences used for qPCR amplification of EST genes from *L. formolongi*. Figure S1: Nucleotide and deduced amino acid sequences of seven stress-related genes in *L. formolongi*. Figure S2: Sequence alignment of three putative *L*. *formolongi* Hsp70 proteins. Figure S3: Sequence alignment of putative *L*. *formolongi* Hsp90 protein. Figure S4: Sequence alignment of putative *L. formolongi* ubiquitin domain containing protein. Figure S5: Sequence alignment of putative *L. formolongi* cytochrome-b5 domain containing protein. Figure S6: Sequence alignment of putative *L. formolongi* RAB domain containing protein. Figure S7: Schematic representation of the motifs identified in the seven putative *L. formolongi* proteins.
