# Supplementary material for: Identification, Characterization and Expression Profiling of Stress-Related Genes in Easter Lily (Lilium formolongi)
Source: Genes (Basel). 2017 Jun 27;8(7):172. doi: 10.3390/genes8070172 (PMC5541305; doi:10.3390/genes8070172)
Supplement: Supplementary file 1 [file genes-08-00172-s001.zip › supplementary/Supplementary figure.docx]

> KX683998


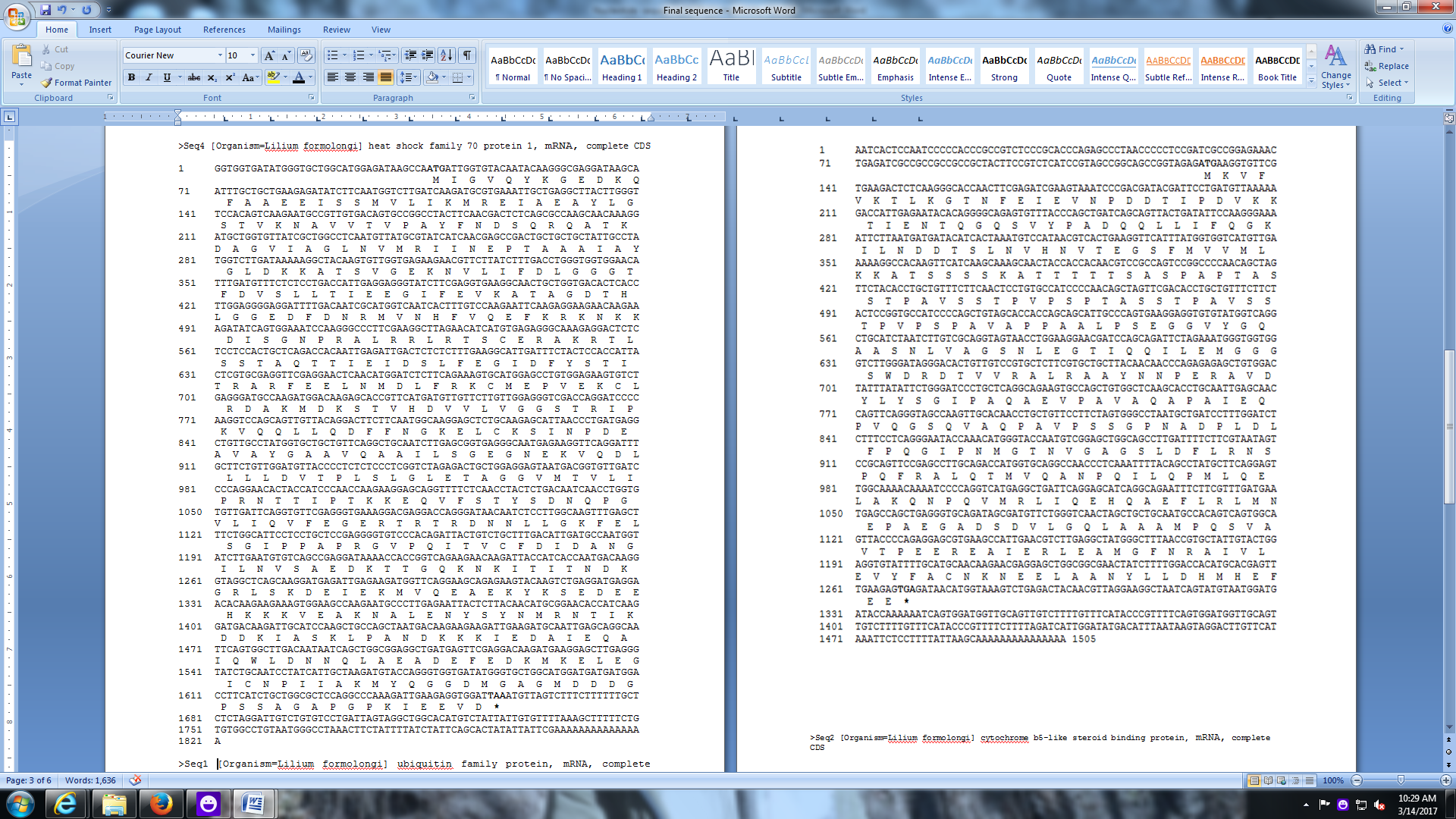


> KX683999


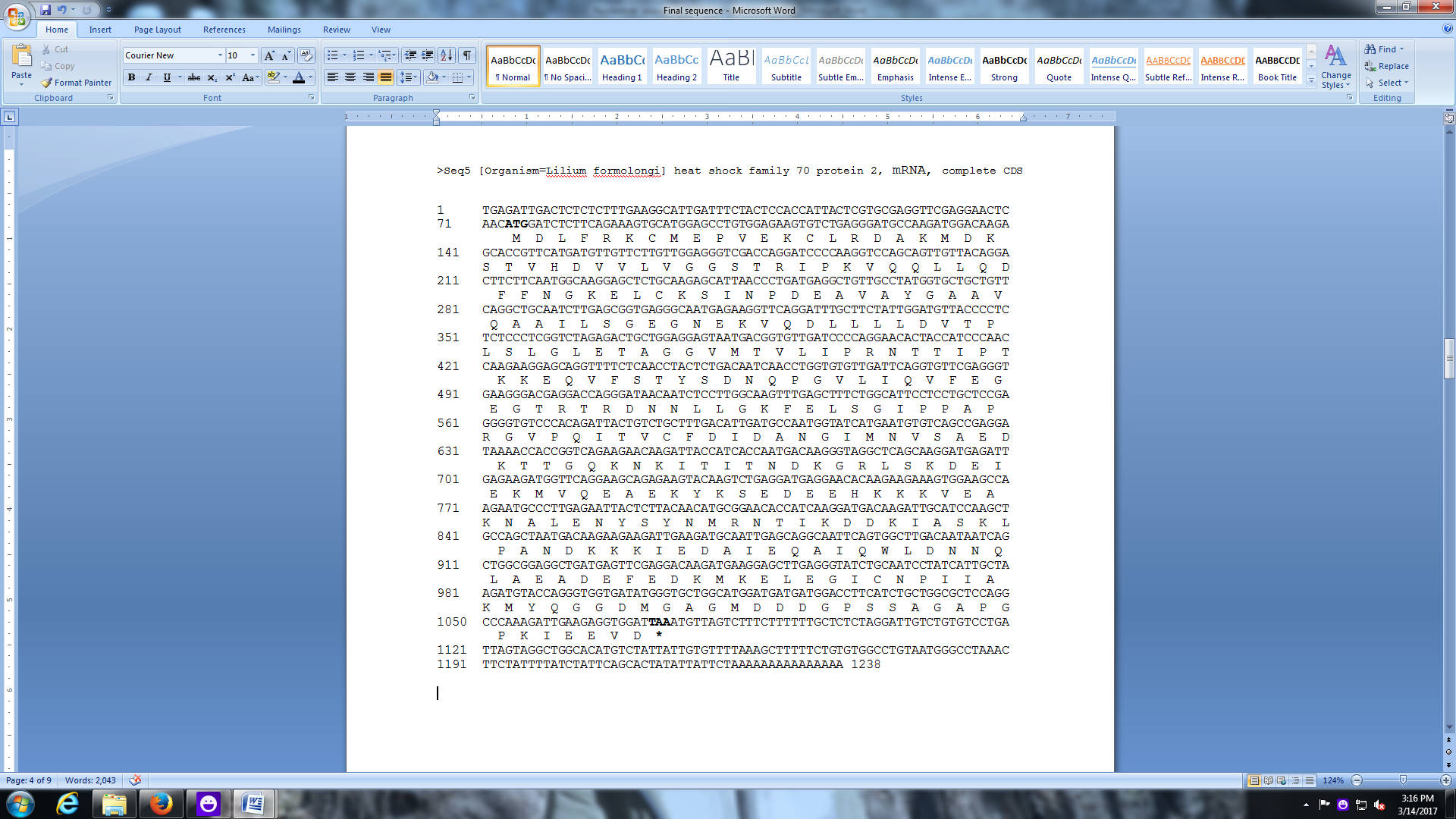


> KX684000


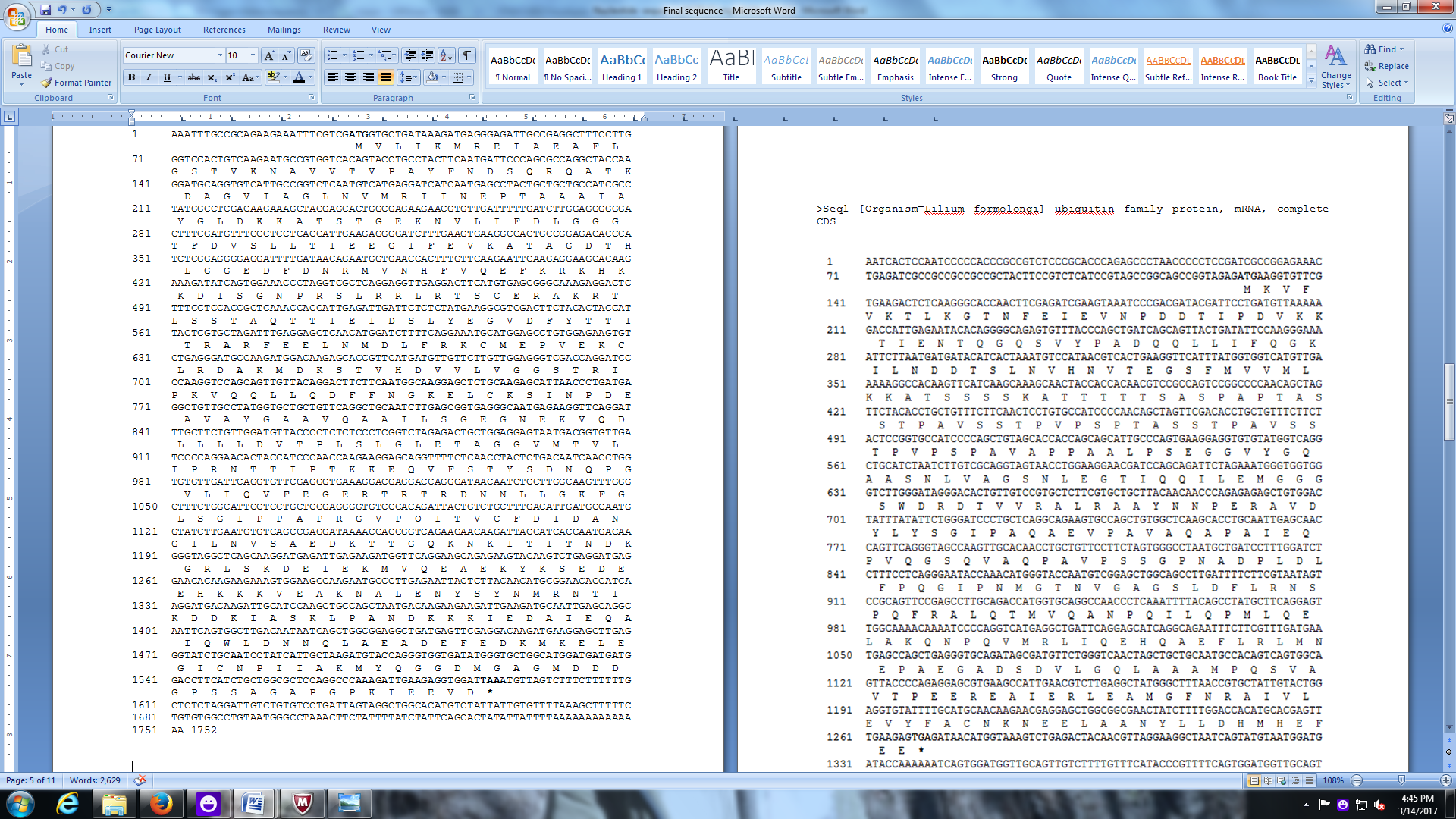


>KX684001


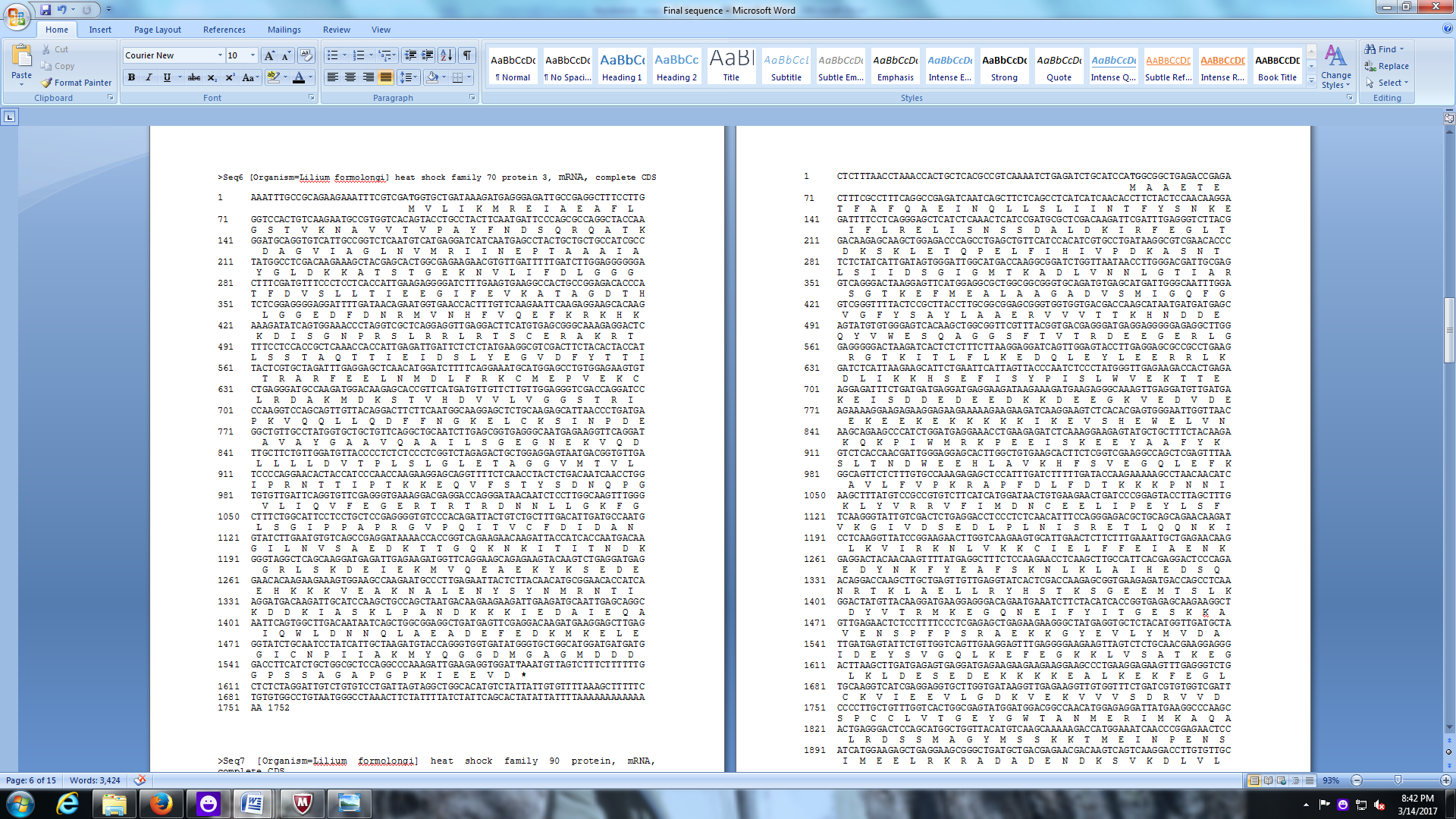


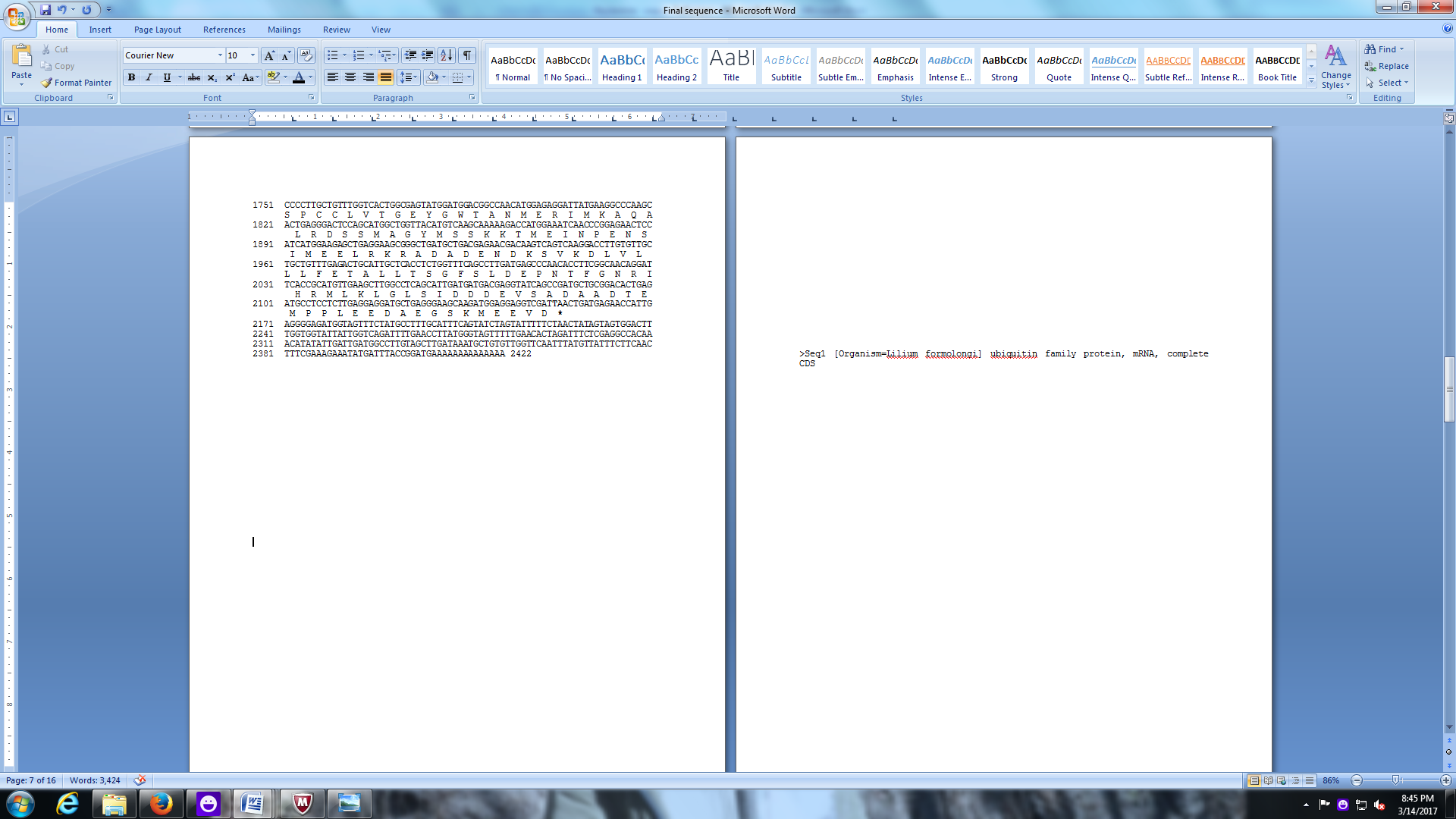


>KX683995


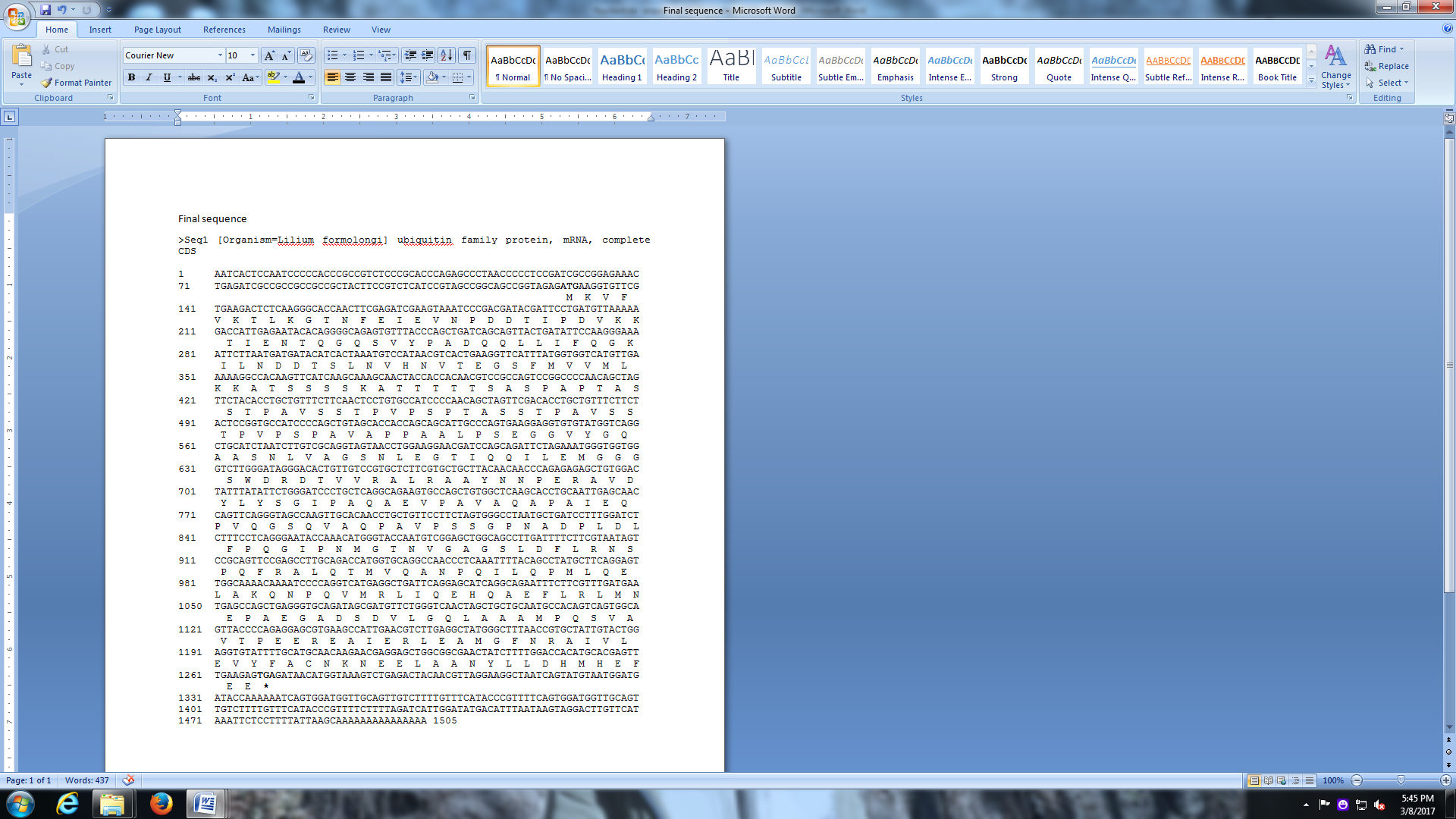


>KX683996


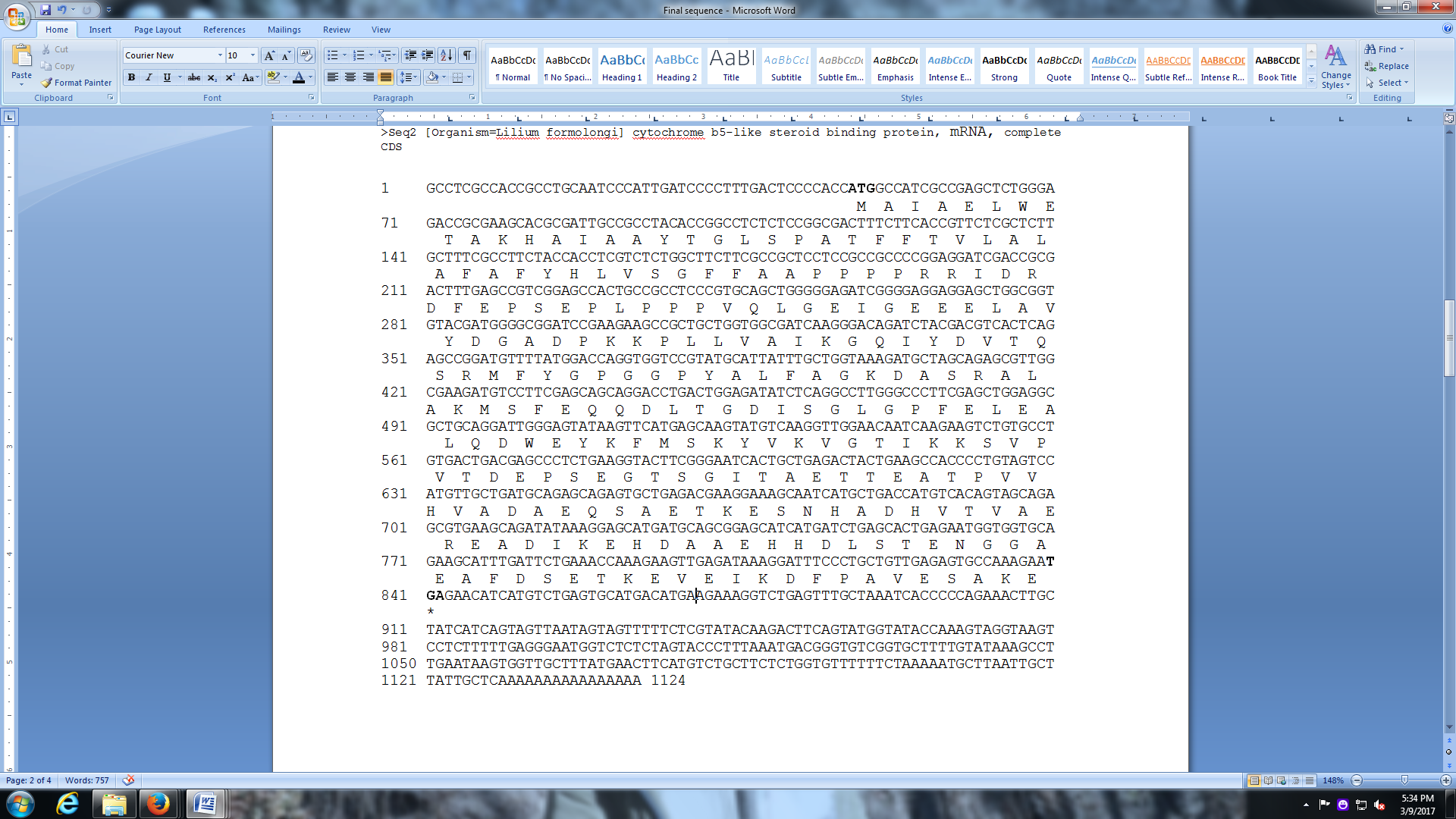


> KX683997


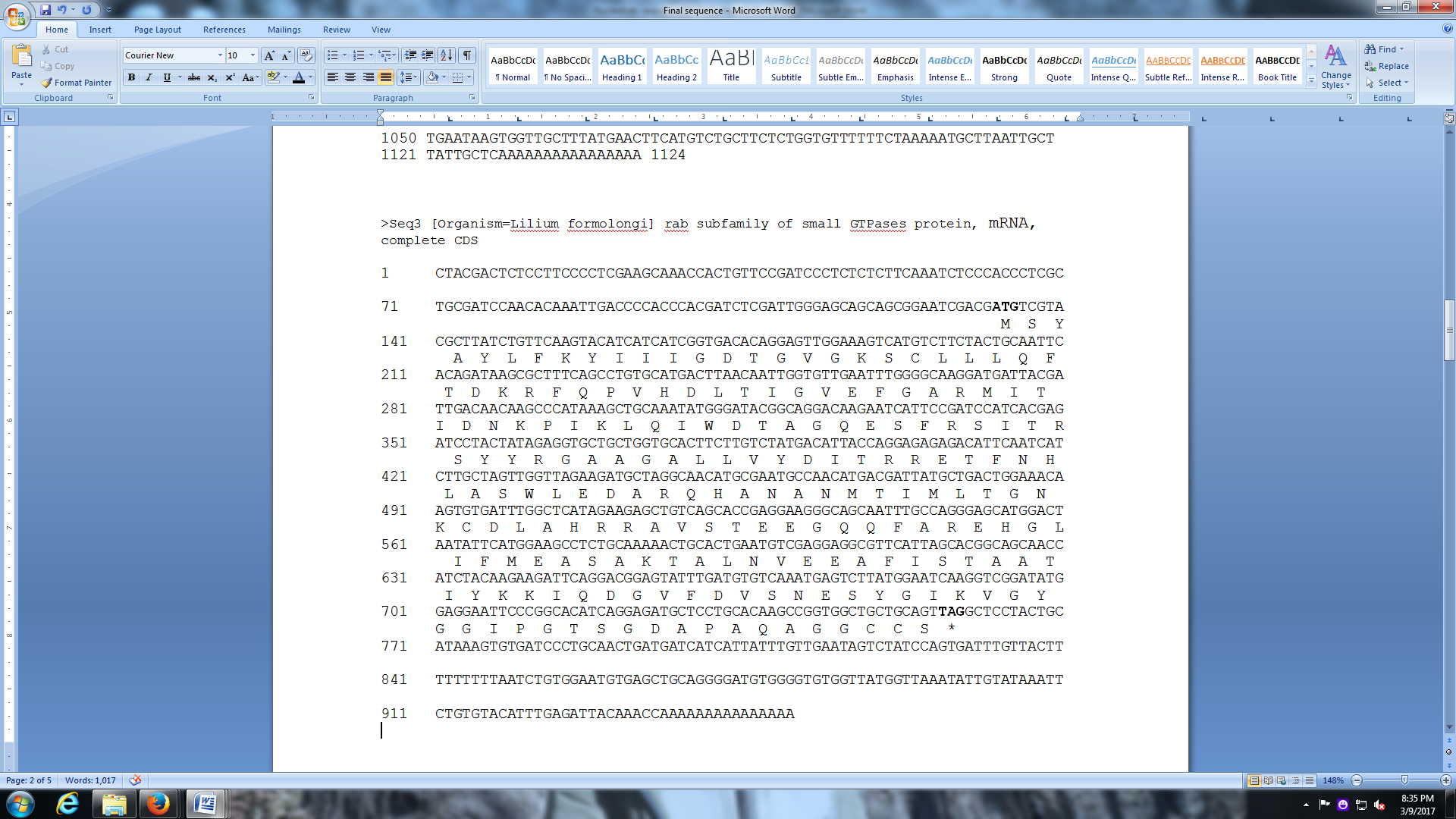


Figure S1: Nucleotide and deduced amino acid sequences of seven stress-related genes (GenBank accessions: KX683995–KX684001) in *L*. *formolongi*. The deduced amino acid sequence is indicated in one-letter code. The start and stop codons (asterisks) are indicated in bold font.

Figure S2: Sequence alignment of three putative *L*. *formolongi* Hsp70 proteins (indicated in colored bold font) using Jalview program. Conservation of amino acids is shaded in different colors. Solid red colored box indicates the highly conserved Hsp70 domain position. The conserved ATPase, and peptide-binding domain followed by substrate-binding site are indicated by lines above the amino acid residues at the N-terminal and C-terminal region, respectively [45,81]. The abbreviated protein names are enlisted in Table 1 and Table S4.

Figure S3: Sequence alignment of putative *L*. *formolongi* Hsp90 protein (indicated in colored bold font) using Jalview program. Conservation of amino acids is shaded in different colors. Solid blue and red boxes indicate the highly conserved HATPase_c and Hsp90 domains position, respectively. The conserved phosphorylation motif [96] and the substrate binding site with MEEVD at N- [46] and C-terminal, respectively are indicated by lines above the amino acid residues. Leucine residues in the leucine zipper motif are indicated by vertical lines [96]. The abbreviated protein names are enlisted in Table 1 and Table S4.

Figure S4: Sequence alignment of putative *L. formolongi* ubiquitin domain containing protein (indicated in colored bold font) using Jalview program. Conservation of amino acids is shaded in different colors. Solid blue colored box indicates UBQ domain position at N-terminal position while two UBA domains separated by STI1 domain are indicated by red and black colored boxes, respectively. The abbreviated protein names are enlisted in Table 1 and Table S4.

Figure S5: Sequence alignment of putative *L. formolongi* cytochrome-b5 domain containing protein (indicated in colored bold font) using Jalview program. Conservation of amino acids is shaded in different colors. Solid colored blue and red boxes indicate the conserved TM and Cyt-b5 domain positions, respectively. The abbreviated protein names are enlisted in Table 1 and Table S4.

Figure S6: Sequence alignment of putative *L. formolongi* RAB domain containing protein (indicated in colored bold font) using Jalview. Conservation of amino acids is shaded in different colors. Solid colored boxes indicate highly conserved domain positions. The abbreviated protein names are enlisted in Table 1 and Table S4.

Figure S7: Schematic representation of the motifs identified in the seven putative *L. formolongi* proteins (indicated in colored bold font). Different motifs are indicated by different colors, along with their respective widths. The common names of the reference proteins and the seven putative proteins identified in this study are listed in Table 1 and Table S4.
