# Supplementary material for: Identification, Characterization and Expression Profiling of Stress-Related Genes in Easter Lily (Lilium formolongi)
Source: Genes (Basel). 2017 Jun 27;8(7):172. doi: 10.3390/genes8070172 (PMC5541305; doi:10.3390/genes8070172)
Supplement: Supplementary file 1 [file genes-08-00172-s001.zip › supplementary/Supplementary tables.docx]

|  |  |  |
| --- | --- | --- |

**Table S1.** Nucleotide homology analysis of 12 EST genes from *Lilium formolongi*^a^

| ^b^EST Gene Name | Gene Name | NCBI Accession No. (EST) | Top Homologous Clones | Name of Protein | Identity (%) | E-value | Top Homologous Species | References |
| --- | --- | --- | --- | --- | --- | --- | --- | --- |
| *Lf1* | - | BP176836 | AK426987.1 | mRNA | 77 | 2e-73 | *Brachypodium distachyon* | [53] |
| *Lf2* | - | BP176893 | AY224432.1 | DNAJ like protein | 75 | 7e-63 | *Oryza sativa* | [54] |
| *Lf3* | - | BP176925 | EU541356.1 | Heat shock protein 70 (Hsp70) | 83 | 1e-88 | *Dactylis glomezrata* | [55] |
| *Lf4* | - | BP176980 | AF076253.1 | Calcineurin B-like protein 3 | 83 | 1e-142 | *Arabidopsis thaliana* | [56] |
| *Lf5* | - | BP177055 | - | - | - |  | *-* | - |
| *Lf6* | *LfHsp70-1* | BP177099 | NM001202918.1 | Heat shock protein 70-3 | 79 | 2e-123 | *Arabidopsis thaliana* | [57] |
| *Lf7* | *LfHsp70-2* | BP177242 | L26243.1 | Heat shock C70 protein | 81 | 1e-114 | *Spinacia oleracea* | [58] |
| *Lf8* | *LfHsp70-3* | BP177599 | NM112093.2 | Heat shock protein 70-4 | 78 | 2e-72 | *Arabidopsis thaliana* | [57] |
| *Lf9* | *LfHsp90* | BP177462 | S77849.1 | Heat shock protein 81-3 | 80 | 8e-102 | *Arabidopsis thaliana* | [59] |
| *Lf10* | *LfUb* | BP176996 | [XM009419379.1](https://www.ncbi.nlm.nih.gov/nucleotide/695058664?report=genbank&log$=nucltop&blast_rank=1&RID=ZT4MMA42014) | ubiquitin receptor RAD23d-like | 82 | 4e-32 | *Musa acuminata* | Unpublished |
| *Lf11* | *LfCyt-b5* | BP177031 | S94464.1 | Polyubiquitin | 85 | 2e-168 | *Zea mays* | [60] |
| *Lf12* | *LfRab* | BP177058 | XM007049388.1 | RAB GTPase B1C | 82 | 1e-125 | *Theobroma cacao* | [61] |

^a^Analyzed using BLAST from NCBI, <http://www.ncbi.nlm.nih.gov/BLAST/>.^b^Expressed sequence tag (EST)

**Table S2.** Specific ligated primer sequences used for 3′ RACE PCR amplification of seven stress-related genes from *L. formolongi*

| ^*^EST-Gene Name | Gene Name | Gene Name | Forward Primer (5´-3´) |
| --- | --- | --- | --- |
| *Lf6* | *LfHsp70-1* | *L. formolongi heat shock 70 protein 1* | CTGATCTAGAGGTACCGGATCCCCTGACCATTGAGGAGGGTA |
| *Lf7* | *LfHsp70-2* | *L. formolongi heat shock 70 protein 2* | CTGATCTAGAGGTACCGGATCCCTTGAATGTGTCAGCCGAGG |
| *Lf8* | *LfHsp70-3* | *L. formolongi heat shock 70 protein 3* | CTGATCTAGAGGTACCGGATCCCCTCCACTGCTCAGACTACA |
| *Lf9* | *LfHsp90* | *L. formolongi heat shock 90 protein* | CTGATCTAGAGGTACCGGATCCCCTTTGGGTAGAGAAGACCT |
| *Lf10* | *LfUb* | *L. formolongi ubiquitin family protein* | CTGATCTAGAGGTACCGGATCCAGTGAAGGAGGTGTGTATGG |
| *Lf11* | *LfCyt-b5* | *L. formolongi cytochrome-b5 protein* | CTGATCTAGAGGTACCGGATCCGAAGATGAGTCGCTGCTGGT |
| *Lf12* | *LfRab* | *L. formolongi RAB protein* | CTGATCTAGAGGTACCGGATCCCACGATCTCGATTGGGAGCA |

**Table S3.** Specific primer sequences used for cDNA synthesis and 5′ RACE PCR amplification of seven stress-related genes from *L. formolongi*

|  |  | First PCR primer set | | Second PCR Primer Set | |
| --- | --- | --- | --- | --- | --- |
| Gene Name | Phosphorylated RT Primer for cDNA Synthesis | Forward Primer (F1) (5´-3´) | Reverse Primer (R1) (5´-3´) | Forward Primer (F2) (5´-3´) | Reverse Primer (R2) (5´-3´) |
| *LfHsp70-1* | AGTCCTCTTTGC | GCTGCTATTGCCTATGGTCT | TGACCTTAAAAGGCCAGAGC | AGGCTACAAGTGTTGGTGAG | CCTGGACAGATGCATCACTG |
| *LfHsp70-2* | CATCATCATCCATGC | GCTTGAGGGTATCTGCAATC | TGTGTTCCTCATCCTCAGAC | CTAAGATGTACCAGGGTGGT | TCTCTGCTTCCTGAACCATC |
| *LfHsp70-3* | CTGGTACATCTTAGC | GAGCAGGCAATTCAGTGGCT | GAACGGTGCTCTTGTCCATC | AGGCTGATGAGTTCGAGGAC | CTTCTCCACAGGCTCCATGC |
| *LfHsp90* | GAGCACCTCATAGC | ACTCGACCAAGAGCGGTGAA | CCACTCGTGTGAGACTTCCT | AGGCTGTTGAGAACTCTCCT | ATCCTCAACTTTGCCCTCTT |
| *LfUb* | GTGCATGTGGTCCA | AGCGTGAAGCCATTGAACGT | GTAAGCAGCACGAAGAGCAC | GCATGCAACAAGAACGAGGA | AGTGTCCCTATCCCAAGACC |
| *LfCyt-b5* | CACTCTCAACAGCA | GTAGCAGAGCGTGAAGCAGA | TTCGCCAACGCTCTGCTAGC | CTGAGAATGGTGGTGCAGAA | TCCCTTGATCGCCACCAGCA |
| *LfRab* | TCTCCTGATGTG | CTGCACTGAATGTCGAGGAG | GCTGAAAGCGCTTATCTGTG | CAAGGTCGGATATGGAGGAA | CCAACTCCTGTGTCACCGAT |

|  |  |
| --- | --- |

**Table S4.** Protein homology analysis of stress-related genes in *L. formolongi*

| EST name | Protein name | NCBI top matched clones | | | | Identity (%) | E-value | References |
| --- | --- | --- | --- | --- | --- | --- | --- | --- |
|  |  | Accession | Homologous species | Types of protein | Abbreviated name |  |  |  |
| Lf6 | LfHsp70-1 | NP195870.1 | *Arabidopsis thaliana* | Heat shock cognate protein 70-1 | AtHsp70-1 | 93 | 0 | Direct submission |
|  |  | ABG22609.1 | *Oryza sativa Japonica Group* | Heat shock cognate protein 70 | OsjHsp70 | 94 | 0 | [62] |
|  |  | CAA47948.2 | *Oryza sativa Indica Group* | Heat shock protein 70 | OsiHsp70 | 94 | 0 | [63] |
|  |  | XP006378714.1 | *Populus trichocarpa* | Heat shock cognate protein 70 | PtHsp70) | 93 | 0 | [64] |
|  |  | XP007027052.1 | *Theobroma cacao* | Heat shock cognate 70 protein-1 isoform-1 | TcHsp70-1 | 94 | 0 | [61] |
| Lf7 | LfHsp70-2 | ABG22609.1 | *Oryza sativa Japonica Group* | Heat shock cognate protein 70 | OsjHsp70 | 92 | 0 | [62] |
|  |  | XP006378714.1 | *Populus trichocarpa* | Heat shock cognate protein 70 | PtHsp70 | 92 | 0 | [64] |
|  |  | CAA47948.2 | *Oryza sativa Indica Group* | Heat shock protein 70 | OsiHsp70 | 92 | 0 | [63] |
|  |  | XP00362 5487.2 | *Medicago truncatula* | Heat shock cognate 70 protein | MtHsp70 | 92 | 0 | [65] |
|  |  | XP007027052.1 | *Theobroma cacao* | Heat shock cognate 70 protein-1 isoform-1 | TcHsp70-1-1 | 93 | 0 | [61] |
| Lf8 | LfHsp70-3 | XP007027052.1 | *Theobroma cacao* | Heat shock cognate 70 protein-1 isoform-1 | TcHsp70-1-1 | 94 | 0 | [61] |
|  |  | \|  \| \| --- \|   CAA47948.2 | *Oryza sativa Indica Group* | Heat shock protein 70 | OsiHsp70 | 93 | 0 | [63] |
|  |  | ABG22609.1 | *Oryza sativa Japonica Group* | Heat shock cognate protein 70 | OsjHsp70 | 92 | 0 | [62] |
|  |  | XP006378714.1 | *Populus trichocarpa* | Heat shock cognate protein 70 | PtHsp70 | 93 | 0 | [64] |
| Lf9 | LfHsp90 | BAD04054.1 | *Oryza sativa Japonica Group* | Heat shock protein 90 | OsjHsp90 | 91 |  | Direct submission |
|  |  | ADF31757.1 | *Triticum aestivum* | Heat shock protein 90 | TaHsp90 | 90 | 0 | [66] |
|  |  | NP001234439.1 | *Solanum lycopersicum* | Heat shock cognate protein 80 | SlHsp80 | 91 | 0 | [67] |
|  |  | NP001275844.1 | *Citrus sinensis* | Heat shock protein 90 | CsHsp90 | 91 | 0 | [68] |
|  |  | BAG16518.1 | *Capsicum chinense* | Heat shock protein 90-2 | CacHsp90-2 | 90 | 0 | Unpublished |
| Lf10 | LfUb | GAV75661.1 | *Cephalotus follicularis* | ubiquitin domain-containing protein | CfUb | 67 | 3e-176 | [69] |
|  |  | XP006383464.1 | *Populus trichocarpa* | Ubiquitin family protein | PtUb | 66 | 2e-169 | [64] |
|  |  | XP_020269018.1 | *Asparagus officinalis* | Low quality protein: ubiquitin receptor RAD23d-like | AoRad23d-like | 67 | 2e-176 | NCBI database |
|  |  | XP008775144.1 | *Phoenix dactylifera* | Predicted: ubiquitin receptor RAD23d-like | PdRad23d-like | 70 | 0 | Unpublished |
|  |  | XP010916168.1 | *Elaeis guineensis* | Predicted: ubiquitin receptor RAD23c-like | EgRad23c-like | 72 | 0 | Unpublished |
| Lf11 | LfCyt-b5 | [XP002321894.1](http://www.ncbi.nlm.nih.gov/protein/224134737?report=genbank&log$=prottop&blast_rank=22&RID=CNCTJPY4014) | *Populus trichocarpa* | Cytochrome b5 protein | PtCytb5 | 73 | 1e-88 | [64] |
|  |  | NP00115018.1 | *Zea mays* | Membrane steroid-binding protein 1 | ZmMsbp-1 | 73 | 2e-79 | [70] |
|  |  | NP_190458.1 | Arabidopsis thaliana | membrane-associated progesterone binding protein 3 | AtMapbp3 | 67 | 1e-90 | [71] |
|  |  | OAP06864.1 | [Arabidopsis thaliana] | MSBP2 | AtMsbp2 | 70 | \| 2e-90 \|  \| \| --- \| --- \| | [72] |
|  |  | OAY82576.1 | Ananas comosus | membrane steroid-binding protein 2 | AcMsbp2 | 57 | 4e-87 | Unpublished |
|  |  | XP_020239868.1 | Cajanus cajan | membrane steroid-binding protein 2-like | CcMsbp | 68 | 1e-89 | NCBI database |
| Lf12 | LfRab | NP193450.1 | *Arabidopsis thaliana* | RABGTPase homolog B1C | AtRabGTPase | 93 | \| 6e-143 \|  \| \| --- \| --- \| | [73] |
|  |  | XP_020080367.1 | *Ananas comosus* | ras-related protein RABB1c | AcRabB1 | 92 | 2e-142 | NCBI databse |
|  |  | XP_010096501.1 | Morus notabilis | Ras-related protein | MnRas | 93 | 3e-143 | Direct submission |
|  |  | KMZ75600.1 | *Zostera marina* | Ras-related protein RABB1c | ZmRas | 91 | 8e-140 | [74] |
|  |  | GAV70579.1 | *Cephalotus follicularis* | Ras domain-containing protein | CfRas | 97 | 3e-137 | [69] |
|  |  | EOX93607.1 | Theobroma cacao | RAB GTPase B1C | TcRabGTPaseB1 | 89 | 8e-136 | [61] |
|  |  | OMO89586.1 | *Corchorus olitorius* | Small GTPase superfamily | CoGTPase | 87 | 2e-135 | Unpublished |

**Table S5.** Nucleotide sequence relatedness among the seven stress-related genes from *L. formolongi**

| EST-Gene Name | Gene Name | a | b | c | d | e | f | g |
| --- | --- | --- | --- | --- | --- | --- | --- | --- |
| *Lf6* | *LfHsp70-1 (a)* | 100 |  |  |  |  |  |  |
| *Lf7* | *LfHsp70-2 (b)* | 99 | 100 |  |  |  |  |  |
| *Lf8* | *LfHsp70-3 (c)* | 92 | 98 | 100 |  |  |  |  |
| *Lf9* | *LfHsp90 (d)* | 22 | 24 | 24 | 100 |  |  |  |
| *Lf10* | *LfUb (e)* | 23 | 24 | 22 | 22 | 100 |  |  |
| *Lf11* | *LfCyt-b5 (f)* | 25 | 24 | 24 | 25 | 24 | 100 |  |
| *Lf12* | *LfRab (g)* | 22 | 21 | 20 | 24 | 21 | 23 | 100 |

^*^ Results from pairwise nucleotide sequence comparisons (ClustalW) are shown as percent identity among the seven genes investigated.

**Table S6.** Specific primer sequences used for qRT-PCR amplification of EST genes from *L. formolongi*

| EST-Gene Name | Gene Name | Forward Primer (5´-3´) | Reverse Primer (5´-3´) | Melting Temperature  (°C) | Product Size (bp) |
| --- | --- | --- | --- | --- | --- |
| *Lf6* | *LfHsp70-1* | GTGAAATTGCTGAGGCTTAC | CCAACACTTGTAGCCTTTTT | 58 | 196 |
| *Lf7* | *LfHsp70-2* | TGCTTCTATTGGATGTTACC | GCTGACACATTCATGATAC | 58 | 298 |
| *Lf8* | *LfHsp70-3* | GGACTTTCGATGTTTCCCTC | AACCTCCTGAGCGACCTAGG | 58 | 181 |
| *Lf9* | *LfHsp90* | TTGGGAGGGGGACTAAGATCA | TCCTCATCCTCATCATCAGA | 58 | 172 |
| *Lf10* | *LfUb* | AAAGCAACTACCACCACAAC | CCATACACACCTCCTTCACT | 58 | 182 |
| *Lf11* | *LfCyt-b5* | GATCGACCGCGACTTTGAGC | CCAGCAAATAATGCATACGGAC | 61 | 198 |
| *Lf12* | *LfRab* | GCAAGGATGATTACGATTGAC | AGCATCTTCTAACCAACTAGC | 58 | 180 |
|  | *Lf-actin* | ATGGAACTGGAATGGTTAAG | ATAGCAACATACATAGCAGG | 58 | 373 |
